# Bioinformatic evaluation of L-arginine catabolic pathways in 24 cyanobacteria and transcriptional analysis of genes encoding enzymes of L-arginine catabolism in the cyanobacterium *Synechocystis *sp. PCC 6803

**DOI:** 10.1186/1471-2164-8-437

**Published:** 2007-11-28

**Authors:** Sarah Schriek, Christian Rückert, Dorothee Staiger, Elfriede K Pistorius, Klaus-Peter Michel

**Affiliations:** 1 Lehrstuhl für Molekulare Zellphysiologie, Universität Bielefeld, Universitätsstr. 25, D-33615 Bielefeld, Germany; 2 Lehrstuhl für Genetik, Universität Bielefeld, Universitätsstr. 25, D-33615 Bielefeld, Germany

## Abstract

**Background:**

So far very limited knowledge exists on L-arginine catabolism in cyanobacteria, although six major L-arginine-degrading pathways have been described for prokaryotes. Thus, we have performed a bioinformatic analysis of possible L-arginine-degrading pathways in cyanobacteria. Further, we chose *Synechocystis *sp. PCC 6803 for a more detailed bioinformatic analysis and for validation of the bioinformatic predictions on L-arginine catabolism with a transcript analysis.

**Results:**

We have evaluated 24 cyanobacterial genomes of freshwater or marine strains for the presence of putative L-arginine-degrading enzymes. We identified an L-arginine decarboxylase pathway in all 24 strains. In addition, cyanobacteria have one or two further pathways representing either an arginase pathway or L-arginine deiminase pathway or an L-arginine oxidase/dehydrogenase pathway. An L-arginine amidinotransferase pathway as a major L-arginine-degrading pathway is not likely but can not be entirely excluded. A rather unusual finding was that the cyanobacterial L-arginine deiminases are substantially larger than the enzymes in non-photosynthetic bacteria and that they are membrane-bound. A more detailed bioinformatic analysis of *Synechocystis *sp. PCC 6803 revealed that three different L-arginine-degrading pathways may in principle be functional in this cyanobacterium. These are (i) an L-arginine decarboxylase pathway, (ii) an L-arginine deiminase pathway, and (iii) an L-arginine oxidase/dehydrogenase pathway. A transcript analysis of cells grown either with nitrate or L-arginine as sole N-source and with an illumination of 50 μmol photons m^-2 ^s^-1 ^showed that the transcripts for the first enzyme(s) of all three pathways were present, but that the transcript levels for the L-arginine deiminase and the L-arginine oxidase/dehydrogenase were substantially higher than that of the three isoenzymes of L-arginine decarboxylase.

**Conclusion:**

The evaluation of 24 cyanobacterial genomes revealed that five different L-arginine-degrading pathways are present in the investigated cyanobacterial species. In *Synechocystis *sp. PCC 6803 an L-arginine deiminase pathway and an L-arginine oxidase/dehydrogenase pathway represent the major pathways, while the L-arginine decarboxylase pathway most likely only functions in polyamine biosynthesis. The transcripts encoding the enzymes of the two major pathways were constitutively expressed with the exception of the transcript for the carbamate kinase, which was substantially up-regulated in cells grown with L-arginine.

## Background

L-arginine metabolism is more complex than the majority of other metabolic pathways in living organisms. This is due to (1) the occurrence of a biosynthetic branch point at the level of carbamoylphosphate, a precursor for L-arginine and pyrimidine biosynthesis, (2) the fact that L-arginine is a potential precursor of polyamines, (3) the fact that L-arginine can be a precursor of 4-aminobutyrate, having a role as neurotransmitter in mammals, (4) the function of L-arginine as a precursor for nitric oxide, acting as an abundant signal molecule in bacteria, mammals, and plants, and (5) the existence of an impressive variety of L-arginine-degrading pathways in eubacteria and archaea. Compared to heterotrophically-growing prokaryotes, L-arginine has specific additional roles in cyanobacteria, because some strains have an alternative carbon dioxide fixation pathway with carbamoylphosphate as the first carbon dioxide fixation product. This pathway leads to the formation of L-citrulline and subsequently to L-arginine [1,2]. Moreover, a number of cyanobacteria is able to synthesize the polymer cyanophycin (multi-L-arginyl-poly-L-aspartate), which consists of an aspartic acid backbone with L-arginine residues being attached to the β-carboxyl group of aspartate by isopeptide bonds [3-6]. Cyanophycin has been shown to have a complex dynamic metabolism, which is not yet completely understood [6-12].

**Figure 1:**
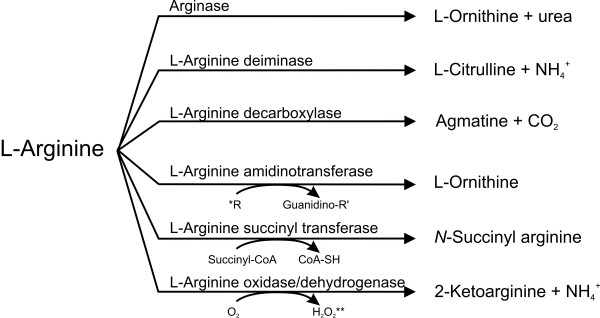
**Six major L-arginine-degrading pathways have been described in bacteria**. The first enzymatic reaction of each pathway is shown. *Transfer of an amidino group to an acceptor such as glycine, L-lysine or inosamine phosphate. **Molecular oxygen or other electron acceptors such as NADP^+ ^or quinones.

L-Arginine serves as a source of nitrogen, carbon, and energy through a variety of catabolic pathways in archaea and eubacteria [13-16]. In eubacteria, six major L-arginine-degrading pathways have been described (Fig. 1). The first enzymes of these six pathways are an arginase, an L-arginine deiminase, an L-arginine decarboxylase, an L-arginine amidino-transferase, an L-arginine succinyl transferase, and an L-arginine oxidase/dehydrogenase, respectively. Heterotrophically growing bacteria contain either only one of these pathways or have multiple catabolic pathways, as e.g. shown for several *Pseudomonas *species [13,14]. In *Pseudomonas putida *and *Pseudomonas aeruginosa *four L-arginine-degrading pathways are functional. The L-arginine succinyl transferase pathway and the L-arginine deiminase pathway serve as major routes of L-arginine catabolism under aerobic and anaerobic conditions, respectively. In addition, an L-arginine oxidase/dehydrogenase pathway also contributes to L-arginine catabolism under aerobic conditions. The role of a fourth pathway, the L-arginine decarboxylase pathway, still remains somewhat unclear. Although it may provide ammonium from L-arginine, it does not seem to play a major role in L-arginine utilization as carbon source. It may have its major function in the biosynthesis of the polyamines agmatine and putrescine [16].

The understanding of cyanobacterial L-arginine catabolism is scarce and only a few studies on L-arginine-degrading enzymes exist. This work includes the detection of arginase and L-arginine deiminase activity in *Anabaena cylindrica *(being synonymous with *Nostoc *sp. PCC 7120 and *Anabaena *sp. PCC 7120) [17], *Anabaena variabilis *[18], *Aphanocapsa *PCC 6308 [19], and *Nosto*c sp. PCC 73102 [20]. In *Synechocystis *sp. PCC 6803 two genes encoding ureohydrolase-type enzymes (Sll1077 and Sll0228) have been identified using bioinformatic tools [21]. L-Ornithine was detected as a major initial product of L-arginine degradation. Based on the detected products, a model of L-arginine catabolism with a putative arginase as the first enzyme has been proposed [21]. In this model L-arginine degradation via arginase is suggested to lead to L-ornithine as first product and subsequently to the production of L-glutamate, and also L-proline. Since L-citrulline and a minor amount of argininosuccinate were also detected as products, an urea cycle-type pathway, besides an arginase pathway, was included in the model [21].

In the two closely related strains *Synechococcus elongatus *PCC 6301 and PCC 7942 an L-amino acid oxidase (AoxA) with a high specificity for basic L-amino acids and with L-arginine as preferred substrate has been partially characterized [22-24]. Recently, such an enzyme has also been identified by enzymatic activity tests in *Synechococcus cedrorum *PCC 6908 [23]. The *aoxA *genes in *Synechococcus elongatus *PCC 6301 and PCC 7942 have also been identified [23].

Since L-arginine catabolism in heterotrophically growing eubacteria is very diverse and since the knowledge on L-arginine catabolism in cyanobacteria is rather limited, the genomes of 24 cyanobacterial strains were screened for the presence of genes encoding putative L-arginine-degrading enzymes in order to obtain an overview on L-arginine catabolism in cyanobacteria. We chose *Synechocystis *sp. PCC 6803 as a model organism and validated the results of our bioinformatic analysis for this strain with a transcript analysis. We chose *Synechocystis *sp. PCC 6803, because results on the products of L-arginine degradation have been published more recently [21].

## Results and Discussion

### Evaluation of 24 cyanobacterial genomes for the presence of genes encoding enzymes of L-arginine-degrading pathways

We used a bioinformatic approach to analyze 24 cyanobacterial strains with fully sequenced and annotated genomes for the presence of genes encoding putative enzymes being involved in the degradation of L-arginine. Among the marine cyanobacteria, the genomes of six *Prochlorococcus *and six *Synechococcus *species as well as the genomes of two N_2_-fixing species (*Crocosphaera watsonii *WH 8501 and *Trichodesmium erythraeum *IMS 101) were investigated. The investigated freshwater cyanobacteria included three mesophilic strains, *Synechococcus elongatus *PCC 6301, *Synechococcus elongatus *PCC 7942, and *Synechocystis *sp. PCC 6803, and three thermophilic strains, *Thermosynechococcus elongatus *BP-1, and two *Synechococcus *Yellowstone species. The latter two thermophilic strains are capable of N_2_-fixation with a diurnal rhythm. Moreover, three heterocyst-forming N_2_-fixing species *Anabaena variabilis *ATCC 29413, *Nostoc *sp. PCC 7120, and *Nostoc punctiforme *PCC 73102 as well as *Gloeobacter violaceus *PCC 7421, a strain which lacks thylakoid membranes, were investigated. The origins of the evaluated cyanobacterial genome sequences are listed in Table 1. Sequences of genes encoding enzymes involved in L-arginine degradation in various archaea and heterotrophically growing eubacteria were used to identify corresponding genes in cyanobacteria (Table 2). The results of the bioinformatic analyses of the 24 cyanobacterial genomes are given in Tables 3 and 4.

**Table 1:** Origin of the 24 cyanobacterial genome sequences that were used to perform the bioinformatic evaluation of the presence of L-arginine-degrading pathways in cyanobacteria.

**Cyanobacterial strain**	Origin of genome sequence*	**Reference sequence**	**GenBank**	**Mbps**	**%GC**	**Proteins/RNAs**
**Marine species**						

*Prochlorococcus marinus *SS 120	European Union/Genoscope	NC_005042	AE017126	1.75	36.4	1883/46
*Prochlorococcus marinus *MIT 9211	Craig Venter Institute	NZ_AALP00000000	AALP00000000	1.84	39.7	2123/45
*Prochlorococcus marinus *MIT 9312	JGI/MIT/DOE	NC_007577	CP000111	1.71	31.2	1810/45
*Prochlorococcus marinus *MIT9313	JGI/DOE	NC_005071	BX548175	2.41	50.7	2269/55
*Prochlorococcus marinus *MED 4	JGI/DOE	NC_005072	BX548174	1.70	30.8	1717/44
*Prochlorococcus marinus *NATL 2A	JGI/MIT/DOE	NC_007335	CP000095	1.84	35.1	1892/44
*Synechococcus *sp. WH 8102	JGI/DOE	NC_005070	BX548020	2.44	59.4	2519/55
*Synechococcus *sp. CC 9902	JGI/DOE	NC_007513	CP000097	2.24	54.2	2307/51
*Synechococcus *sp. RS 9917	Craig Venter Institute	NZ_AANP00000000	AANP00000000	2.58	64.5	2770/50
*Synechococcus *sp. CC 9605	JGI/DOE	NC_007516	CP000110	2.51	59.2	2645/54
*Synechococcus *sp. WH 5701	Craig Venter Institute	NZ_AANO00000000	AANO00000000	3.04	65.4	3346/55
*Synechococcus *sp. WH 7805	Craig Venter Institute	NZ_AAOK00000000	AAOK00000000	2.62	57.6	2883/51
*Trichodesmium erythraeum *IMS 101	WHOI/JGI/DOE	NC_008312	CP000393	7.75	34.1	4451/48
*Crocosphaera watsonii *WH 8501	WHOI/JGI/DOE	NZ_AADV00000000	AADV00000000	6.24	37.1	5958/38

**Freshwater species**						

*Synechococcus elongatus *PCC 6301	Nagoya University	NC_006576	AP008231	2.70	55.5	2527/55
*Synechococcus elongatus *PCC 7942	JGI/Texas A & M University/DOE	NC_007604	CP000100	2.70	55.5	2612/53
*Synechocystis *sp. PCC 6803	Kazusa DNA Research Institute	NC_000911	BA000022	3.57	47.7	3172/50
*Gloeobacter violaceus *PCC 7421	Kazusa DNA Research Institute	NC_005125	BA000045	4.66	62.0	4430/52
*Nostoc *sp. PCC 7120	Kazusa DNA Research Institute	NC_003272	BA000019	6.41	41.3	5366/64
*Nostoc punctiforme *PCC 73102	JGI/DOE	NZ_AAAY00000000	AAAY00000000	9.02	41.4	7672/n.d.
*Anabaena variabilis *ATCC 29413	Missouri State University/JGI/DOE	NC_007413	CP000117	6.37	41.4	5043/62
*Thermosynechococcus elongatus *BP-1	Kazusa DNA Research Institute	NC_004113	BA000039	2.59	53.9	2476/49
*Synechococcus *Yellowstone A JA-3-3Ab	TIGR	NC_007775	CP000239	2.93	60.2	2760/55
*Synechococcus *Yellowstone B JA-2-3B'a (2–13)	TIGR	NC_007776	CP000240	3.05	58.5	2862/52

*JGI, Joint Genome Research Institute; DOE, Department of Energy USA; WHOI, Woods Hole Oceanographic Institute; MIT, Massachusetts Institute of Technology; TIGR, The Institute for Genomic Research. The strain *Prochlorococcus marinus *SS 120 corresponds to *Prochlorococcus marinus *subsp. *marinus *str. CCMP 1375 and strain *Prochlorococcus marinus *MED 4 corresponds to *Prochlorococcus marinus *subsp.*pastoris *str. CCMP 1986 or CCMP 1378. *Nostoc *sp. PCC 7120 is synonymous to *Anabaena *sp. PCC 7120 as well as *Anabaena cylindrica*. N.d. = not detected.

**Table 2:** Origin of archaea, eubacterial, and eukaryotic genome sequences used as a reference for the bioinformatic analysis of putative L-arginine-degrading pathways in cyanobacteria.

**Organism**	**Origin of genome sequence**	**Reference sequence**	**GenBank**	**Mbps**	**% GC**	**Number of Proteins/RNA**
**Eubacteria**						

*Escherichia coli *K-12 MG1655	University of Wisconsin-Madison, U.S.A.; *Escherichia coli *Genome Project	NC_000913	U00096	4.64	50.8	4243/157
*Pseudomonas aeruginosa *PAO1	PathoGenesis Corporation, Skokie, U.S.A.;	NC_002516	AE004091	6.30	66.6	5568/81
*Pseudomonas fluorescens *Pf-5	DOE Joint Genome Institute, U.S.A.	NC_004129	CP000076	7.08	63.3	6137/87
*Pseudomonas syringae *pv. *syringae *B728a	DOE Joint Genome Institute, U.S.A.	NC_007005	CP000075	6.09	59.2	5089/83
*Bacillus subtilis *subsp. *subtilis *str. 168	Non-redundant *B. subtilis *database	NC_000964	AL009126	4.22	43.5	4105/119
*Bacillus clausii *KSM-K16	Kao Corporation, Biological Science Laboraties, Japan	NC_006582	AP006627	4.30	44.8	4096/96
*Bacillus halodurans *C-125	Extreme Biosphere Research Center MSTC, Japan	NC_002570	BA000004	4.20	43.7	4066/105
*Xanthomonas campestris *pv. *campestris *str. ATCC 33913	Sao Paulo (State) Consortium	NC_003902	AE008922	5.08	65.1	4181/61
*Corynebacterium glutamicum *ATCC 13032	Kitasato University, Kitasato, Japan	NC_003450	BA000036	3.31	53.8	2993/81
*Brucella melitensis *16M	Integrated Genomics Inc., Chicago, U.S.A.	NC_003317(chr. I)	AE008917	2.12	57.2	2059/48
		NC_003318 (chr. II)	AE008918	1.18	57.3	1139/18
*Ralstonia solanacearum *GMI 1000	Genoscope, Evry cedex, France	NC_003295 (chr.)	AL646052	3.72	67.0	3440/67
		NC_003296 (plas.)	AL646053	2.10	66.9	1676/7

**Higher Plants**						

*Arabidopsis thaliana (thale cress)*	*Arabidopsis *Genome Initiative	NC_003070 (chr. 1)	AE005172	30.43	35.7	7852/7852
		NC_003071 (chr. 2)	AE002093	19.71	35.9	4853/4853
		NC_003074 (chr. 3)	BA000014	23.47	36.3	6048/6048
		NC_003075 (chr. 4)	AJ270058	18.58	36.2	4655/4655
		NC_003076 (chr. 5)	BA000015	26.99	35.9	7072/7072

A sequence from *Synechococcus *sp. Yellowstone B JA-2-3B'a 2–13 was used to screen for L-arginine amidinotransferase sequences. The screen for L-arginine oxidase/dehydrogenases was performed with the *aoxA *sequence from *Synechococcus elongatus *PCC 6301/PCC 7942.

**Table 3:** Presence of genes encoding enzymes of the L-arginine-degrading pathways in the genomes of selected marine and freshwater cyanobacteria.

**Pathway**	**L-Arginine decarboxylase**							
**Enzymes**	**A1**	**A2.1**	**A2.2**	**A2.3**	**A3**	**A4**	**A5**	**A6**

**Marine species**								

*Prochlorococcus marinus *SS 120	+	+	n.d.	+	n.d.	+	+	+
*Prochlorococcus marinus *str. MIT 9211	+	+	n.d.	+	n.d.	+	+	+
*Prochlorococcus marinus *MIT 9312	+	+	n.d.	+	n.d.	+	+	+
*Prochlorococcus marinus *MIT 9313	+	+	n.d.	+	n.d.	+	+	+
*Prochlorococcus marinus *MED 4	+	+	n.d.	+	n.d.	+	+	+
*Prochlorococcus marinus *NATL 2A	+	+	n.d.	+	n.d.	+	+	+
*Synechococcus *sp. CC 9605	+	+	n.d.	+	n.d.	+	+	+
*Synechococcus *sp. CC 9902	+	+	n.d.	+	n.d.	+	+	+
*Synechococcus *sp. WH 8102	+	+	n.d.	+	n.d.	+	+	+
*Synechococcus *sp. WH 7805	+	+	n.d.	+	n.d.	n.d.	+	+
*Synechococcus *sp. WH 5701	+	+	n.d.	+	n.d.	+	+	+
*Synechococcus *sp. RS 9917	+	+	n.d.	+	n.d.	+	+	+
*Crocosphaera watsonii *WH 8501	+	n.d.	n.d.	+	n.d.	+	+	+
*Trichodesmium erythraeum *IMS 101	+	+	n.d.	+	n.d.	+	+	+

**Freshwater species**								

*Synechococcus elongatus *sp. PCC 6301	+	n.d.	+	+	n.d.	+	+	+
*Synechococcus elongatus *sp. PCC 7942	+	n.d.	+	+	n.d.	+	+	+
*Synechococcus Yellowstone *sp. A JA-3-3-AB	+	+	n.d.	+	n.d.	+	+	+
*Synechococcus Yellowstone *sp. B JA-2-3B'a (2–13)	+	+	n.d.	+	n.d.	+	+	+
*Thermosynechococcus elongatus *BP-1	+	n.d.	+	+	n.d.	+	+	+
*Synechocystis *sp. PCC 6803	+	+	n.d.	+	n.d.	+	+	+
*Gloeobacter violaceus *PCC 7421	+	n.d.	+	+	n.d.	+	+	+
*Nostoc *sp. PCC 7120	+	+	n.d.	+	n.d.	+	+	+
*Nostoc punctiforme *PCC 73102	+	+	n.d.	+	n.d.	+	+	+
*Anabaena variabilis *ATCC 29413	+	+	n.d.	+	n.d.	+	+	+

**Figure 2:**
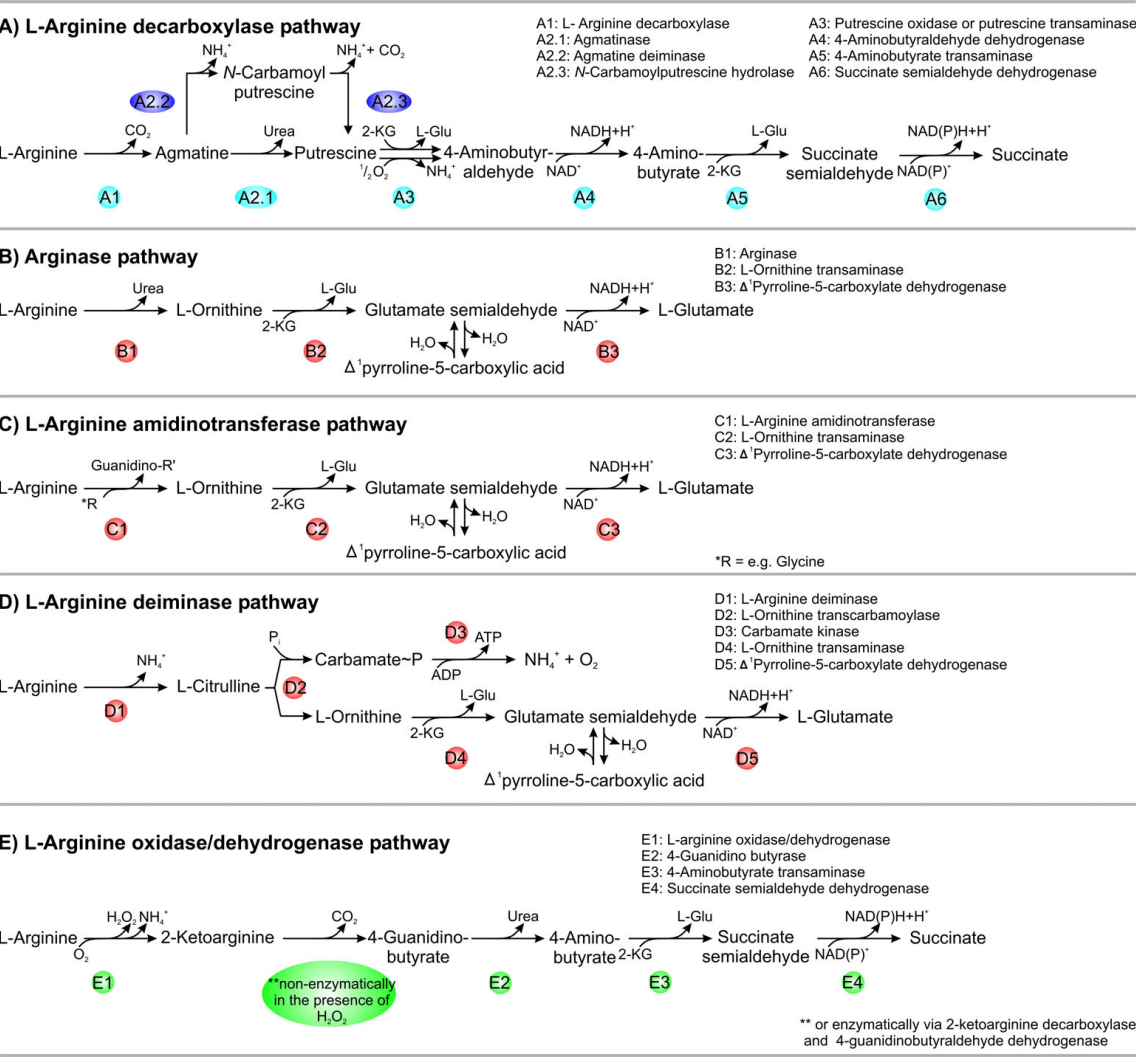
**Schematic presentation of putative L-arginine-degrading pathways in cyanobacteria with the corresponding enzymes, intermediate metabolites, and final products**. Numbering of enzymes refers to the one used in Table 3, 4, and 5–9.

L-ornithine is formed from L-arginine by the enzymes arginase or L-arginine amidinotransferase. It is also formed in the 2^nd ^reaction of the L-arginine deiminase pathway. Enzymes A5 and E3 are identical enzymes and both represent a 4-aminobutyrate transaminase. Enzymes A6 and E4 are identical and both represent a succinate semialdehyde dehydrogenase (Fig. 2). Enzymes A2.1, B1, and C1 represent ureohydrolases, and the same gene(s) is (are) annotated as an agmatinase (A2.1), an arginase (B1) or a 4-guanidinobutyrase (E2). The genes encoding the enzymes C1 and D1 are annotated as L-arginine amidinotransferase as well as L-arginine deiminase (see text for further details). N.d. = not detected.

**Table 4:** Presence of genes encoding enzymes of the L-arginine-degrading pathways in the genomes of selected marine and freshwater cyanobacteria.

**Pathway**	**Arginase**			**L-Arginine amidinotransferase**			**L-Arginine deiminase**					**L-Arginine oxidase/dehydrogenase**			
**Enzymes**	**B1**	**B2**	**B3**	**C1**	**C2**	**C3**	**D1**	**D2**	**D3**	**D4**	**D5**	**E1**	**E2**	**E3**	**E4**

**Marine species**															

*Prochlorococcus marinus *SS 120	+	+	+	n.d.	+	+	n.d.	+	n.d.	+	+	n.d.	+	+	+
*Prochlorococcus marinus *str. MIT 9211	+	+	+	n.d.	+	+	n.d.	+	n.d.	+	+	n.d.	+	+	+
*Prochlorococcus marinus *MIT 9312	+	+	+	n.d.	+	+	n.d.	+	n.d.	+	+	n.d.	+	+	+
*Prochlorococcus marinus *MIT 9313	+	+	+	n.d.	+	+	n.d.	+	n.d.	+	+	n.d.	+	+	+
*Prochlorococcus marinus *MED 4	+	+	+	n.d.	+	+	n.d.	+	n.d.	+	+	n.d.	+	+	+
*Prochlorococcus marinus *NATL 2A	+	+	+	n.d.	+	+	n.d.	+	n.d.	+	+	n.d.	+	+	+
*Synechococcus *sp. CC 9605	+	+	+	n.d.	+	+	n.d.	+	n.d.	+	+	+	+	+	+
*Synechococcus *sp. CC 9902	+	+	+	n.d.	+	+	n.d.	+	n.d.	+	+	n.d.	+	+	+
*Synechococcus *sp. WH 8102	+	+	+	n.d.	+	+	n.d.	+	n.d.	+	+	n.d.	+	+	+
*Synechococcus *sp. WH 7805	+	+	+	n.d.	+	+	n.d.	+	n.d.	+	+	+	+	+	+
*Synechococcus *sp. WH 5701	+	+	+	n.d.	+	+	n.d.	+	n.d.	+	+	+	+	+	+
*Synechococcus *sp. RS 9917	+	+	+	n.d.	+	+	n.d.	+	n.d.	+	+	n.d.	+	+	+
*Crocosphaera watsonii *WH 8501	n.d.	+	+	+	+	+	+	n.d.	n.d.	+	+	n.d.	n.d.	+	+
*Trichodesmium erythraeum *IMS 101	+	+	+	+	+	+	+	+	n.d.	+	+	+	+	+	+

**Freshwater species**															

*Synechococcus elongatus *sp. PCC 6301	n.d.	+	+	n.d.	+	+	n.d.	+	n.d.	+	+	+	n.d.	+	+
*Synechococcus elongatus *sp. PCC 7942	n.d.	+	+	n.d.	+	+	n.d.	+	n.d.	+	+	+	n.d.	+	+
*Synechococcus Yellowstone *sp. A JA-3-3-AB	+	+	+	n.d.	+	+	n.d.	n.d.	n.d.	+	+	n.d.	+	+	+
*Synechococcus Yellowstone *sp. B JA-2-3B'a (2–13)	+	+	+	+	+	+	+	n.d.	n.d.	+	+	n.d.	+	+	+
*Thermosynechococcus elongatus *BP-1	n.d.	+	+	+	+	+	+	+	n.d.	+	+	n.d.	n.d.	+	+
*Synechocystis *sp. PCC 6803	+	+	+	+	+	+	+	+	+	+	+	+	+	+	+
*Gloeobacter violaceus *PCC 7421	n.d.	+	+	+	+	+	+	+	n.d.	+	+	+	n.d.	+	+
*Nostoc *sp. PCC 7120	+	+	+	+	+	+	+	+	n.d.	+	+	+	+	+	+
*Nostoc punctiforme *PCC 73102	+	+	+	+	+	+	+	+	n.d.	+	+	+	+	+	+
*Anabaena variabilis *ATCC 29413	+	+	+	+	+	+	+	n.d.	n.d.	+	+	n.d.	+	+	+

In total, we found evidence for the presence of five putative pathways for L-arginine catabolism in the investigated genomes. These are an L-arginine decarboxylase pathway, an arginase pathway, an L-arginine amidinotransferase pathway, an L-arginine deiminase pathway, and an L-arginine oxidase/dehydrogenase pathway. These pathways are outlined (Fig. 2), and the accession numbers of the corresponding genes are given as supplement in Tables 5, 6, 7, 8, 9. No evidence has been found for the presence of an L-arginine succinyl transferase pathway.

**Table 5:** Database entries of genes from 24 cyanobacterial genomes encoding putative L-arginine decarboxylases (A1), agmatinases (A2.1), agmatine deiminases (A2.2), *N*-carbamoylputrescine hydrolases (A2.3), putrescine oxidases or putrescine transaminases (A3), and 4-aminobutyraldehyde dehydrogenases (A4) of the L-arginine decarboxylase pathway.

**Enzyme**	**A1**	**A2.1**	**A2.2**	**A2.3**	**A3**	**A4**
**Marine species**						

*Prochlorococcus marinus *SS 120	Pro1112, Pro0049	Pro1849	n.d	Pro1045	n.d	Pro1319
*Prochlorococcus marinus *str. MIT 9211	P9211_03242, P9211_08607	P9211_09067	n.d	P9211_03592	n.d	P9211_07012
*Prochlorococcus marinus *MIT 9312	PMT9312_1095, PMT9312_0046	PMT9312_1779	n.d	PMT9312_0615	n.d	PMT9312_0337
*Prochlorococcus marinus *MIT 9313	PMT1066, PMT2150	PMT2214	n.d	PMT0395	n.d	PMT0191
*Prochlorococcus marinus *MED 4	PMM1084, PMM0045	PMM1686	n.d	PMM0615	n.d	PMM1215, PMM0331
*Prochlorococcus marinus *NATL 2A	PMN2A_0665, PMN2A_1378	PMN2A_1287	n.d	PMN2A_0052	n.d	PMN2A_1709
*Synechococcus *sp. CC 9605	Syncc9605_1621, Syncc9605_2513	Syncc9605_1082Syncc9605_2591	n.d	Syncc9605_1134	n.d	Syncc9605_0497
*Synechococcus *sp. CC 9902	Syncc9902_1380, Syncc9902_2172	Syncc9902_2230	n.d	Syncc9902_1323	n.d	Syncc9902_1838
*Synechococcus *sp. WH 8102	SYNW0944, SYNW2359	SYNW1412, SYNW2422	n.d	SYNW1008	n.d	SYNW_1956
*Synechococcus *sp. WH 7805	WH7805_04481, WH7805_10353	WH7805_09974	n.d	WH7805_01902	n.d	n.d
*Synechococcus *sp. WH 5701	WH5701_04905, WH5701_10310	WH5701_03684, WH5701_03860	n.d	WH5701_10020, WH5701_10155	n.d	WH5701_06196
*Synechococcus *sp. RS 9917	RS9917_01007, RS9917_06495	RS9917_06190	n.d	RS9917_11395	n.d	RS9917_02641
*Crocosphaera watsonii *WH 8501	CwatDRAFT_1880	n.d	n.d	CwatDRAFT_4111	n.d	CwatDRAFT_2611CwatDRAFT_0842 CwatDRAFT_0969 CwatDRAFT_1032
*Trichodesmium erythraeum *IMS 101	TeryDRAFT_0894, TeryDRAFT_0959, TeryDRAFT_0311	TeryDRAFT4567	n.d	TeryDRAFT_0835	n.d	TeryDRAFT_3296, TeryDRAFT_3923

**Freshwater species**						

*Synechococcus elongatus *sp. PCC 6301	Syc0823_d, Syc0510_c	n.d	SYC1703_c, SYC1643_d	Syc1946_d, Syc1745_c	n.d	Syc1030_d
*Synechococcus elongatus *sp. PCC 7942	Synpcc7942_0707, Synpcc7942_1037	n.d	Synpcc79422402 Synpcc79422461	Synpcc79422145 Synpcc79422358	n.d	Synpcc7942_0489
*Synechococcus Yellowstone *sp. JA-3-3-AB	CYA_1002, CYA_0128	CYA_0859	n.d	CYA_0558	n.d	CYA_0364
*Synechococcus Yellowstone sp. JA-2-3Ba (2-13) *	CYB_2779, CYB_0482	CYB_1744	n.d	CYB_1181	n.d	CYB_0715, CYB_1893
*Thermosynechococcus elongatus *BP-1	Tlr1866, Tll1807	n.d.	Tlr0111	Tlr0112, Tll0920	n.d	Tlr0221
*Synechocystis *sp. PCC 6803	Sll1683, Slr0662, Slr1312	Sll1077, Sll0228	n.d	Sll0601, Sll1640	n.d	Sll1495, Slr0370
*Gloeobacter violaceus *PCC 7421	Gll4070, Gll3478	n.d	Glr1681	Glr1682, Glr2043	n.d	Gll2207, Gll1504, Glr3848, Gll2805
*Nostoc *sp. PCC 7120	All3401, All4887	Alr2310	n.d	Alr2001	n.d	Alr2826, Alr3771, All3556, All5022
*Nostoc punctiforme *PCC 73102	Npun02000556, Npun02000612	Npun02002114	n.d	Npun02002053	n.d	Npun02003427, Npun02002895, Npun02002692, Npun02003702
*Anabaena variabilis *ATCC 29413	Ava_2157, Ava_3423	Ava_0127	n.d	Ava_5061	n.d	Ava_1107, Ava_1554, Ava_3534, Ava_2258

N.d. = not detected.

**Table 6:** Database entries of genes from 24 cyanobacterial genomes encoding putative arginases (B1), L-ornithine transaminases (C2), and Δ^1 ^pyrroline-5-carboxylate dehydrogenases (C3) of the arginase pathway.

**Enzyme**	**B1**	**B2**	**B3**
**Marine species**			

*Prochlorococcus marinus *SS 120	Pro1849	Pro1375, Pro1626	Pro0374
*Prochlorococcus marinus *str. MIT 9211	P9211_09067	P9211_02002, P9211_10217	P9211_07012
*Prochlorococcus marinus *MIT 9312	PMT9312_1779	PMT9312_1397, PMT9312_1565	PMT9312_0337
*Prochlorococcus marinus *MIT 9313	PMT2214	PMT0331, PMT1493	PMT0191
*Prochlorococcus marinus *MED 4	PMM1686	PMM1301, PMM1472	PMM0331
*Prochlorococcus marinus *NATL 2A	PMN2A_1287	PMN2A_0867, PMN2A_1003	PMN2A_1709
*Synechococcus *sp. CC 9605	Syncc9605_1082, Syncc9605_2591	Syncc9605_0858, Syncc9605_2052, Syncc9605_0659	Syncc9605_0497
*Synechococcus *sp. CC 9902	Syncc9902_2230	Syncc9902_1534, Syncc9902_0620	Syncc9902_1838
*Synechococcus *sp. WH 8102	SYNW1412, SYNW2422	SYNW1634, SYNW0629	SYNW1956
*Synechococcus *sp. WH 7805	WH7805_06086, WH7805_09974	WH7805_05656, WH7805_12388, WH7805_13803	WH7805_06416
*Synechococcus *sp. WH 5701	WH5701_03684, WH5701_03860	WH5701_07406, WH5701_15376	WH5701_06196
*Synechococcus *sp. RS 9917	RS9917_06190	RS9917_02041, RS9917_05240	RS9917_02641
*Crocosphaera watsonii *WH 8501	n.d.	CwatDRAFT_5161	CwatDRAFT_0865, CwatDRAFT_0842, CwatDRAFT_0969
*Trichodesmium erythraeum *IMS 101	TeryDRAFT_4567	TeryDRAFT_3251	TeryDRAFT_2672 TeryDRAFT_3296, TeryDRAFT_3923

**Freshwater species**			

*Synechococcus elongatus *sp. PCC 6301	n.d.	Syc0599_c, Syc1466_c	Syc1030_d
*Synechococcus elongatus *sp. PCC 7942	n.d.	Synpcc7942_0943, Synpcc7942_0031	Synpcc7942_0489
*Synechococcus Yellowstone *sp. JA-3-3-AB	CYA_0859	CYA_1537, CYA_0689	CYA_0364
*Synechococcus Yellowstone sp. JA-2-3Ba (2-13)*	CYB_1744	CYB_1419, CYB_2128	CYB_0516, CYB_0715, CYB_1893
*Thermosynechococcus elongatus *BP-1	n.d.	Tlr1328, Tlr0408, Tll1935	Tlr0416, Tlr0221
*Synechocystis *sp. PCC 6803	Sll1077, Sll0228	Slr1022	Sll1561, Slr0370, Slr0091
*Gloeobacter violaceus *PCC 7421	n.d.	Glr0547, Glr3849, Gll2223	Glr2755, Glr3848, Gll1504, Gll2805
*Nostoc *sp. PCC 7120	Alr2310	Alr2398, Alr1080, All0396	Alr0540, Alr3771, All3556, All5022
*Nostoc punctiforme *PCC 73102	Npun02002114	Npun02005728, Npun02001164, Npun02001509	Npun02003702, Npun02006572, Npun02002895, Npun02002692
*Anabaena variabilis *ATCC 29413	Ava_0127	Ava_0214, Ava_3730, Ava_2839	Ava_2942, Ava_1554, Ava_3534, Ava_2258

N.d. = not detected.

**Table 7:** Database entries of genes from 24 cyanobacterial genomes encoding putative L-arginine amidinotransferases (C1), L-ornithine transaminases (C2), and Δ^1 ^pyrroline-5-carboxylate dehydrogenases (C3) of the L-arginine amidinotransferase pathway.

**Enzyme**	**C1**	**C2**	**C3**
**Marine species**			

*Prochlorococcus marinus *SS 120	n.d.	Pro1375, Pro1626	Pro0374
*Prochlorococcus marinus *str. MIT 9211	n.d.	P9211_02002, P9211_10217	P9211_07012
*Prochlorococcus marinus *MIT 9312	n.d.	PMT9312_1397, PMT9312_1565	PMT9312_0337
*Prochlorococcus marinus *MIT 9313	n.d.	PMT0331, PMT1493	PMT0191
*Prochlorococcus marinus *MED 4	n.d.	PMM1301, PMM1472	PMM0331
*Prochlorococcus marinus *NATL 2A	n.d.	PMN2A_0867, PMN2A_1003	PMN2A_1709
*Synechococcus *sp. CC 9605	n.d.	Syncc9605_0858, Syncc9605_2052, Syncc9605_0659	Syncc9605_0497
*Synechococcus *sp. CC 9902	n.d.	Syncc9902_1534, Syncc9902_0620	Syncc9902_1838
*Synechococcus *sp. WH 8102	n.d.	SYNW1634, SYNW0629	SYNW1956
*Synechococcus *sp. WH 7805	n.d.	WH7805_05656, WH7805_12388, WH7805_13803	WH7805_06416
*Synechococcus *sp. WH 5701	n.d.	WH5701_07406, WH5701_15376	WH5701_06196
*Synechococcus *sp. RS 9917	n.d.	RS9917_02041, RS9917_05240	RS9917_02641
*Crocosphaera watsonii *WH 8501	CwatDRAFT_0830	CwatDRAFT_5161	CwatDRAFT_0865, CwatDRAFT_0842, CwatDRAFT_0969
*Trichodesmium erythraeum *IMS 101	TeryDRAFT_2282	TeryDRAFT_3251	TeryDRAFT_2672 TeryDRAFT_3296, TeryDRAFT_3923

**Freshwater species**			

*Synechococcus elongatus *sp. PCC 6301	n.d.	Syc0599_c, Syc1466_c	Syc1030_d
*Synechococcus elongatus *sp. PCC 7942	n.d.	Synpcc7942_0943, Synpcc7942_0031	Synpcc7942_0489
*Synechococcus Yellowstone *sp. JA-3-3-AB	n.d.	CYA_1537, CYA_0689	CYA_0364
*Synechococcus Yellowstone* sp. JA-2-3Ba (2-13)	CYB_0250	CYB_1419, CYB_2128	CYB_0516, CYB_0715, CYB_1893
*Thermosynechococcus elongatus *BP-1	Tll0507	Tlr1328, Tlr0408, Tll1935	Tlr0416, Tlr0221
*Synechocystis *sp. PCC 6803	Sll1336	Slr1022	Sll1561, Slr0370, Slr0091
*Gloeobacter violaceus *PCC 7421	Glr1758	Glr0547, Glr3849, Gll2223	Glr2755, Glr3848, Gll1504, Gll2805
*Nostoc *sp. PCC 7120	Alr4495	Alr2398, Alr1080, All0396	Alr0540, Alr3771, All3556, All5022
*Nostoc punctiforme *PCC 73102	Npun02001803	Npun02005728, Npun02001164, Npun02001509	Npun02003702, Npun02006572, Npun02002895, Npun02002692
*Anabaena variabilis *ATCC 29413	Ava_2273	Ava_0214, Ava_3730, Ava_2839	Ava_2942, Ava_1554, Ava_3534, Ava_2258

N.d. = not detected.

**Table 8:** Database entries of genes from 24 cyanobacterial genomes encoding putative L-arginine deiminases (D1), L-ornithine transcarbamoylases (D2), carbamate kinases (D3), L-ornithine transaminases (D4), and Δ^1 ^pyrroline-5-carboxylate dehydrogenases (D5) of the L-arginine deiminase pathway.

**Enzyme**	**D1**	**D2**	**D3**	**D4**	**D5**
**Marine species**					

*Prochlorococcus marinus *SS 120	n.d.	Pro1337, Pro0262	n.d.	Pro1375, Pro1626	Pro0374
*Prochlorococcus marinus *str. MIT 9211	n.d.	P9211_0227, P9211_07567	n.d.	P9211_02002, P9211_10217	P9211_07012
*Prochlorococcus marinus *MIT 9312	n.d.	PMT9312_1357	n.d.	PMT9312_1397, PMT9312_1565	PMT9312_0337
*Prochlorococcus marinus *MIT 9313	n.d.	PMT0379, PMT1807	n.d.	PMT0331, PMT1493	PMT0191
*Prochlorococcus marinus *MED 4	n.d.	PMM1263, PMM0233	n.d.	PMM1301, PMM1472	PMM0331
*Prochlorococcus marinus *NATL 2A	n.d.	PMN2S_0829	n.d.	PMN2A_0867, PMN2A_1003	PMN2A_1709
*Synechococcus *sp. CC 9605	n.d.	Syncc9605_0926, Syncc9605_0292, Syncc9605_2634	n.d.	Syncc9605_0858, Syncc9605_2052, Syncc9605_0659	Syncc9605_0497
*Synechococcus *sp. CC 9902	n.d.	Syncc9902_1482, Syncc9902_2261, Syncc9902_2051	n.d.	Syncc9902_1534, Syncc9902_0620	Syncc9902_1838
*Synechococcus *sp. WH 8102	n.d.	SYNW1586, SYNW2454, SYNW0296	n.d.	SYNW1634, SYNW0629	SYNW1956
*Synechococcus *sp. WH 7805	n.d.	WH7805_05251, WH7805_09779, WH7805_07451	n.d.	WH7805_05656, WH7805_12388, WH7805_13803	WH7805_06416
*Synechococcus *sp. WH 5701	n.d.	WH5701_14691, WH5701_01185	n.d.	WH5701_07406, WH5701_15376	WH5701_06196
*Synechococcus *sp. RS 9917	n.d.	RS_01761, RS_10896, RS_03633	n.d.	RS9917_02041, RS9917_05240	RS9917_02641
*Crocosphaera watsonii *WH 8501	CwatDRAFT_0830	CwatDRAFT_4406, CwatDRAFT_6596	n.d.	CwatDRAFT_5161	CwatDRAFT_0865, CwatDRAFT_0842, CwatDRAFT_0969
*Trichodesmium erythraeum *IMS 101	TeryDRAFT_2282	TeryDRAFT_0921, TeryDRAFT_1912	n.d.	TeryDRAFT_3251	TeryDRAFT_2672 TeryDRAFT_3296, TeryDRAFT_3923

**Freshwater species**					

*Synechococcus elongatus *sp. PCC 6301	n.d.	Syc1592_c, Syc0859_c	n.d.	Syc0599_c, Syc1466_c	Syc1030_d
*Synechococcus elongatus *sp. PCC 7942	n.d.	Syncc7942_2514, Syncc7942_0670	n.d.	Synpcc7942_0943, Synpcc7942_0031	Synpcc7942_0489
*Synechococcus Yellowstone *sp. JA-3-3-AB	n.d.	CYA_2817, CYA_1730	n.d.	CYA_1537, CYA_0689	CYA_0364
*Synechococcus Yellowstone* sp. JA-2-3Ba (2-13)	CYB_0250	CYB_0821, CYB_1917	n.d.	CYB_1419, CYB_2128	CYB_0516, CYB_0715, CYB_1893
*Thermosynechococcus elongatus *BP-1	Tll0507	Tll1106, Tll1558	n.d.	Tlr1328, Tlr0408, Tll1935	Tlr0416, Tlr0221
*Synechocystis *sp. PCC 6803	Sll1336	Sll0902, Slr1476	Sll0573	Slr1022	Sll1561, Slr0370, Slr0091
*Gloeobacter violaceus *PCC 7421	Glr1758	Gll3101, Gll2875	n.d.	Glr0547, Glr3849, Gll2223	Glr2755, Glr3848, Gll1504, Gll2805
*Nostoc *sp. PCC 7120	Alr4495	Alr4907, All1681	n.d.	Alr2398, Alr1080, All0396	Alr0540, Alr3771, All3556, All5022
*Nostoc punctiforme *PCC 73102	Npun02001803	Npun_02004258, Npun_02007755	n.d.	Npun02005728, Npun02001164, Npun02001509	Npun02003702, Npun02006572, Npun02002895, Npun02002692
*Anabaena variabilis *ATCC 29413	Ava_2273	Ava_2197, Ava_1174	n.d.	Ava_0214, Ava_3730, Ava_2839	Ava_2942, Ava_1554, Ava_3534, Ava_2258

N.d. = not detected.

**Table 9:** Database entries of genes from 24 cyanobacterial genomes encoding putative L-arginine oxidase/dehydrogenase (E1), 4-guanidino butyrase (E2), 4-aminobutyrate transaminase (E3), and succinate semialdehyde dehydrogenase (E4) of the L-arginine oxidase/dehydrogenase pathway.

**Enzymes**	**E1**	**E2**	**E3**	**E4**
**Marine species**				

*Prochlorococcus marinus *SS 120	n.d.	Pro1849	Pro1375, Pro0482, Pro1626	Pro0374
*Prochlorococcus marinus *str. MIT 9211	n.d.	P9211_09067	P9211_02002, P9211_06427, P9211_10217	P9211_00350, P9211_07012
*Prochlorococcus marinus *MIT 9312	n.d.	PMT9312_1779	PMT9312_1397, PMT9312_0484, PMT9312_1565	PMT9312_0337
*Prochlorococcus marinus *MIT 9313	n.d.	PMT2214	PMT0331, PMT1296, PMT0103, PMT1493	PMT0191
*Prochlorococcus marinus *MED 4	n.d.	PMM1686	PMM1301, PMM0483, PMM1472	PMM0331
*Prochlorococcus marinus *NATL 2A	n.d.	PMN2A_1287	PMN2A_0867, PMN2A_1816, PMN2A_1003	PMN2A_1709
*Synechococcus *sp. CC 9605	Syncc9605_1906, Syncc9605_0745	Syncc9605_1082, Syncc9605_2591	Syncc9605_0858, Syncc9605_0659, Syncc9605_2052	Syncc9605_0497
*Synechococcus *sp. CC 9902	n.d.	Syncc9902_2230	Syncc9902_1534, Syncc9902_1701, Syncc9902_0620	Syncc9902_1838
*Synechococcus *sp. WH 8102	n.d.	SYNW1412, SYNW2422	SYNW1634, SYNW1809, SYNW0629	SYNW1956
*Synechococcus *sp. WH 7805	WH7805_05376	WH7805_09974	WH7805_05656, WH7805_1303, WH7805_12388	WH7805_06416
*Synechococcus *sp. WH 5701	WH5701_04470	WH5701_03684, WH5701_03860	WH5701_07406, WH5701_10070, WH5701_15376	WH5701_06196
*Synechococcus *sp. RS 9917	n.d.	RS9917_06190	RS9917_02041, RS9917_05240, RS9917_02041, RS9917_09251	RS9917_02641
*Crocosphaera watsonii *WH 8501	n.d.	n.d.	CwatDRAFT_5161, CwatDRAFT_2647	CwatDRAFT_0842, CwatDRAFT_0969, CwatDRAFT_0865, CwatDRAFT_1032
*Trichodesmium erythraeum *IMS 101	TeryDRAFT_0956	TeryDRAFT4567	TeryDRAFT_3251, TeryDRAFT_3173	TeryDRAFT_3296, TeryDRAFT_3923, TeryDRAFT_3248

**Enzymes**	**C1**	**C2**	C3*	C4**

**Freshwater species**				

*Synechococcus elongatus *sp. PCC 6301	Syc0596_c, Syc1144_c	n.d.	Syc0599_c, Syc1466_c, Syc0881_c	Syc1030_d
*Synechococcus elongatus *sp. PCC 7942	Synpcc7942_0946, Synpcc7942_0369	n.d.	Synpcc7942_0943, Synpcc7942_0031, Synpcc7942_0645	Synpcc7942_0489
*Synechococcus Yellowstone *sp. JA-3-3-AB	n.d.	CYA_0859	CYA_1537, CYA_2386, CYA_0689	CYA_0364
*Synechococcus Yellowstone* sp. JA-2-3Ba (2-13)	n.d.	CYB_1744	CYB_1419, CYB_2128, CYB_1012	CYB_1893, CYB_1419, CYB_0715
*Thermosynechococcus elongatus *BP-1	n.d.	n.d.	Tlr0479, Tlr1328, Tlr0408, Tlr1935	Tlr0221, Tlr0416
*Synechocystis *sp. PCC 6803	Slr0782	Sll1077, Sll0228	Slr1022, Sll0017	Slr0370, Slr0091, Sll1561
*Gloeobacter violaceus *PCC 7421	Gll1123	n.d.	Glr3849, Glr0547, Glr0071, Gll2223	Glr3848, Gll1504, Gll2805
*Nostoc *sp. PCC 7120	Alr7169	Alr2310	Alr2398, Alr1080, All0396, Alr3265	Alr3771, All3556, Alr0540, All5022, Alr3672
*Nostoc punctiforme *PCC 73102	Npun02003735	Npun02002114	Npun02005728, Npun02001509, Npun02001164, Npun02002747	Npun02003702, Npun02002895, Npun02002692, Npun02005276
*Anabaena variabilis *ATCC 29413	n.d.	Ava_0127	Ava_0214, Ava_3730, Ava_2839, Ava_4920	Ava_1554, Ava_3534, Ava_2942, Ava_2258, Ava_3615

N.d. = not detected.

### L-arginine decarboxylase pathway

One or several genes encoding L-arginine decarboxylase-type enzymes, which catalyze the formation of agmatine from L-arginine, are present in all investigated cyanobacteria (Fig. 2, Tables 3 and 5). A putative agmatinase that converts agmatine to putrescine and urea is present in nineteen cyanobacterial strains. No such gene was identified in *Crocosphaera watsonii *WH 8501, *Synechococcus elongatus *PCC 6301, *Synechococcus elongatus *PCC 7942, *Thermosynechococcus elongatus *BP-1, and *Gloeobacter violaceus *PCC 7421. These strains, with the exception of *Crocosphaera watsonii *WH 8501, convert agmatine to putrescine via an agmatine deiminase and an *N*-carbamoylputrescine hydrolase. Since in none of the investigated cyanobacteria a putrescine oxidase or a putrescine transaminase encoding gene has been found, we consider the L-arginine decarboxylase pathway to be mainly responsible for the synthesis of the polyamines agmatine and putrescine as well as for production of ammonium from L-arginine. Putrescine can subsequently be converted to spermidine or spermine. Evidence for the utilization of putrescine by γ-glutamylation like in *E. coli *[25] was not found. However, since transaminases frequently show broad substrate specificity, we can not entirely exclude that a rather unspecific transaminase, which is not annotated as a putrescine transaminase, catalyzes the conversion of putrescine to 4-aminobutyr aldehyde. The subsequent dehydrogenase that converts the aldehyde to 4-aminobutyrate is present in 23 of the 24 investigated strains. Such an enzyme is absent in *Synechococcus *sp. WH 7805. The two enzymes, which catalyze the conversion of 4-aminobutyrate to succinate (4-aminobutyrate transaminase and succinate semialdehyde dehydrogenase) are present in all 24 strains. However, since 4-aminobutyrate also is an intermediate of the L-amino oxidase/dehydrogenase pathway and can additionally be formed by decarboxylation of L-glutamate, the presence of genes encoding the latter two enzymes not necessarily implies that a complete L-arginine decarboxylase pathway is present. Therefore, the question whether the L-arginine decarboxylase pathway only provides polyamines and ammonium or also allows for utilization of L-arginine as C-source can not be answered on the basis of the bioinformatic considerations.

**Figure 3:**
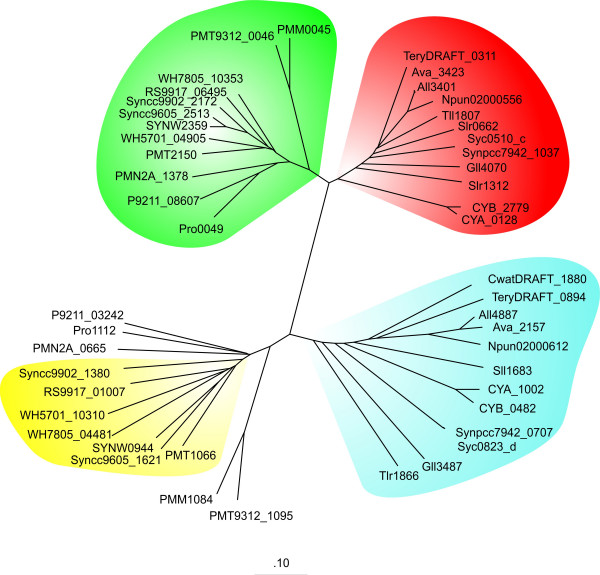
**Phylogenetic tree of cyanobacterial L-arginine decarboxylases**. The L-arginine decarboxylases are the same as in Table 3 and 5.

A phylogenetic tree of the L-arginine decarboxylases, which are present in the investigated cyanobacterial genomes, is given (Fig. 3) and shows that the cyanobacterial L-arginine decarboxylases cluster into four distinct groups. The clusters marked in green and yellow exclusively contain L-arginine decarboxylases of the marine non-N_2_-fixing strains, while the red and blue clusters contain L-arginine decarboxylases of freshwater cyanobacteria and of the two marine N_2_-fixing species *Crocosphaera watsonii *and *Trichodesmium erythraeum *IMS101. It should be pointed out that in species with more than several L-arginine decarboxylase(s) the corresponding enzymes always group into two different clusters. Thus, the marine as well as the fresh water cyanobacteria seem to have two distinct types of L-arginine decarboxylases.

It has previously been shown by Sandmeier et al. [26] that amino acid decarboxylases in general can be subdivided into four different groups. These groups seem to be evolutionary unrelated to each other. In these subdivisions, the groups III and IV contain decarboxylases with specificity for basic L-amino acids. In addition, there is evidence that *E. coli *has two different L-arginine decarboxylases – a biosynthetic and a biodegradable form. The biodegradable L-arginine decarboxylase (P28629 – group III decarboxylase) is only induced in large amounts when cells are grown in rich medium containing L-arginine, while the biosynthetic enzyme (P21170 – group IV decarboxylase) is expressed constitutively [26,27]. On the basis of this classification, the red and green clusters (Fig. 3) contain L-arginine decarboxylases being more similar to group IV L-arginine decarboxylases, while the blue and yellow clusters contain L-arginine decarboxylases with higher similarity to group III L-arginine decarboxylases. The similarity of the biodegradable and the biosynthetic L-arginine decarboxylase of *E. coli *to selected marine and fresh water cyanobacterial L-arginine decarboxylases is presented in Table 10. E.g. the L-arginine decarboxylases Slr0662 and Slr1312 of *Synechocystis *sp. PCC 6803 in the red cluster have a higher similarity to the biosynthetic L-arginine decarboxylase (P21170) of group IV than to the biodegradable L-arginine decarboxylase P28629 of group III. In contrast, Sll1683 of *Synechocystis *sp. PCC 6803 has a higher similarity to P28629 (group III) than to P21170 (group IV) (Table 10). Thus, it is likely that the green and the red cluster (Fig. 3) contain L-arginine decarboxylases of the biosynthetic-type, while the yellow and blue clusters contain L-arginine decarboxylases of the biodegradative type.

**Table 10:** Biochemical properties of selected L-arginine decarboxylases of freshwater and marine cyanobacteria, and their similarity to L-arginine decarboxylases from *E. coli*.

**Strain**	**Database entry**	**AA**	**MM (kDa)**	**pI**	**Group III decarboxylase: ***E. coli *P28629 (biodegradable type) 755 aa; 84.4 kDa; pI 5.12	**Group IV decarboxylase: ***E. coli *P21170 (biosynthetic type) 658 aa; 73.9 kDa; pI 4.83
					**Score vs. P28629**	**Score vs. P21170**

**Yellow cluster decarboxylases**						

*Synechococcus *sp. RS9917	RS9917_01007	470	50.4	9.64	19	8
*Prochlorococcus marinus *str. NATL2A	PMN2A_0665	464	51.5	8.57	11	5
*Prochlorococcus marinus *SS120	Pro1112	440	48.5	5.32	19	5
*Synechococcus *sp. WH 8102	SYNW0994	468	50.6	6.95	18	10

**Blue cluster decarboxylases**						

*Synechocystis *sp. PCC 6803	Sll1683	483	51.8	5.44	24	8
*Gloeobacter violaceus *PCC 7120	Gll3487	467	49.4	6.39	25	8
*Thermosynechococcus elongatus *BP-1	Tlr1866	437	46.6	5.22	22	5
*Anabaena variabilis *ATCC 2941	Ava_2157	488	52.0	5.34	26	7

**Green cluster decarboxylases**						

*Prochlorococcus marinus *MED4	PMM0045	488	50.01	5.34	3	32
*Prochlorococcus marinus *str. MIT 9313	PMT2150	648	71.3	5.31	7	35
*Prochlorococcus marinus *SS120	Pro0049	648	72.4	6.44	3	32
*Synechococcus *sp. WH 7805	WH7805_10353	636	69.9	5.24	7	36
*Prochlorococcus marinus *str. MIT 9211	P9211_08607	648	72.2	6.00	4	33

**Red cluster decarboxylases**						

*Synechocystis *sp. PCC 6803	Slr0662 Slr1312	695 659	78.2 74.5	5.08 5.30	4 4	38 36
*Nostoc *sp. PCC 7120	All3401	671	75.7	5.25	9	37
*Anabaena variabilis *ATCC 2941	Ava_3423	671	75.7	5.25	9	37
*Gloeobacter violaceus *PCC 7120	Gll4070	644	72.7	5.10	7	38

P28629 represents a biodegradable and inducible L-arginine decarboxylase (group III); P21270 represents a biosynthetic and constitutively expressed L-arginine decarboxylase (group IV) in *E. coli *[26]. Score values were calculated with the ClustalW software [62]. The L-arginine decarboxylases in the yellow, blue, green, and red cluster are identical to those L-arginine decarboxylases given in Fig. 3.

### Arginase pathway

**Figure 4:**
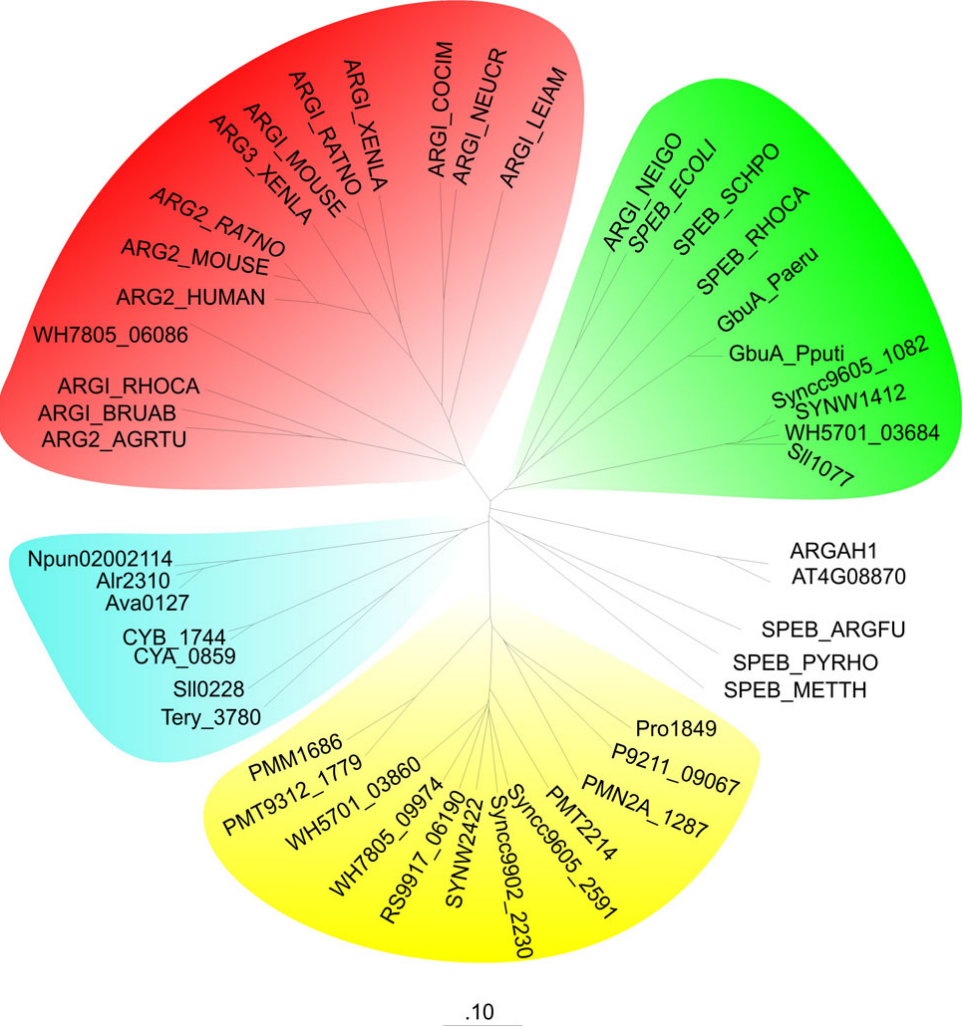
**Phylogenetic tree of ureohydrolases**. For construction of the tree, selected sequences from eubacteria, fungi, plants, and animals were used in addition to the cyanobacterial sequences given (Tables 3 and 4). For details on the non-cyanobacterial sequences see Sekowska et al. [37] and Chen et al. [28]. Details on the cyanobacterial sequences are given (Tables 5, 6, and 9).

Urea is released from L-arginine by an arginase in the arginase pathway, and the resulting L-ornithine is further catabolized to L-glutamate by L-ornithine transaminase and Δ^1^pyrroline-5-carboxylate dehydrogenase (Fig. 2). In the presence of urease, urea is further degraded to ammonium. The arginase pathway seems to be widely distributed among the investigated cyanobacteria. Genes encoding the putative second and third enzyme of this pathway, the L-ornithine transaminase and the Δ^1^pyrroline-5-carboxylate dehydrogenase, are present in all 24 investigated cyanobacteria. A gene encoding a putative arginase is only present in 19 of the investigated genomes (Tables 4 and 6). Such a gene is absent in *Crocosphaera watsonii *WH 8501, *Synechococcus elongatus *PCC 6301, *Synechococcus elongatus *PCC 7942, *Thermosynechococcus elongatus *BP-1, and *Gloeobacter violaceus *PCC 7421. The likely absence of an arginase-type enzyme in five of the investigated 24 cyanobacterial strains is somewhat surprising, since arginases have been shown to be present in all so far investigated higher plants [28]. However, since plant-type arginases represent a distinct group of ureohydrolases [28] (Fig. 4, ARGAH1 and AT4G08870) and localize in mitochondria [29], they may have originated from the predecessor organism, which gave rise to the evolutionary lineage of mitochondria.

### L-arginine amidinotransferase pathway

In addition to arginases, L-ornithine may also be synthesized by L-arginine amidinotransferases (Fig. 2). A gene for such an enzyme was detected in the N_2_-fixing species *Nostoc *sp. PCC 7120, *Nostoc punctiforme *PCC 73102, *Anabaena variabilis *ATCC 29413, *Trichodesmium erythraeum *IMS 101, *Crocosphaera watsonii *WH 8501, *Synechococcus Yellowstone *sp. JA-2-3Ba' (2–13), and in the non-N_2 _fixing cyanobacteria *Synechocystis *sp. PCC 6803, *Thermosynechococcus elongatus *BP-1, and *Gloeobacter violaceus *PCC 7421 (Table 4 and 7). Three of the five cyanobacteria without an arginase-type enzyme have a putative L-arginine amidinotransferase-type enzyme (*Crocosphaera watsonii *WH 8501, *Thermosynechococcus elongatus *BP-1, and *Gloeobacter violaceus *PCC 7421). Thus, *Synechococcus elongatus *PCC 6301 and PCC 7942 are probably the only cyanobacterial strains among the 24 investigated ones, which are unable to form L-ornithine from L-arginine. Interestingly, they have a very active L-amino acid oxidase (AoxA) with high specificity for basic amino acids and a preference for L-arginine, utilizing molecular oxygen as an electron acceptor [22-24].

### L-arginine deiminase pathway

The L-arginine deiminase pathway is widely distributed among eubacteria and archaea [13,14,16] and has also been discovered in a few primitive eukaryotes, e.g. in *Giardia intestinalis *[30], *Trichomonas vaginalis *[31], and *Tritrichomonas foetus *[32]. However, it has so far not been detected in multi-cellular organisms. The L-arginine deiminase pathway consists of three enzymes and catalyzes the production of ATP in its final enzymatic step. The first enzyme of this pathway is an L-arginine deiminase, which irreversibly converts L-arginine to L-citrulline and ammonium. The second and third enzymes are an L-ornithine transcarbamoylase and a carbamate kinase, respectively (Fig. 2). A gene encoding a putative L-arginine deiminase was detected in the N_2_-fixing species *Nostoc *sp. PCC 7120, *Nostoc punctiforme *PCC 73102, *Anabaena variabilis *ATCC 29413, *Trichodesmium erythraeum *IMS 101, *Crocosphaera watsonii *WH 8501, and *Synechococcus Yellowstone *sp. JA-2-3Ba' 2–13 as well as in the non-N_2 _fixing cyanobacteria *Synechocystis *sp. PCC 6803, *Thermosynechococcus elongatus *BP-1, and *Gloeobacter violaceus *PCC 7421 (Tables 4 and 8). This gene is the same as the one being annotated encoding a putative L-arginine amidinotransferase (see below for discussion of this aspect). An L-ornithine transcarbamoylase is present in all investigated cyanobacteria. Since the majority of the investigated cyanobacteria have two genes encoding a putative L-ornithine transcarbamoylase, it is likely that they contain a catabolic and an anabolic enzyme [13,33]. Surprisingly, a carbamate kinase, which catalyzes the last step of the deiminase pathway, has only been detected in *Synechocystis *sp. PCC 6803. An L-arginine deiminase activity has previously been detected in *Anabaena cylindrica *[17], *Anabaena variabilis *[18], *Nostoc *sp. PCC 73102 [20], and *Aphanocapsa *PCC 6308 [19].

### L-arginine oxidase/dehydrogenase pathway

The fifth putative L-arginine catabolic pathway starts with an L-arginine oxidase/dehydrogenase-type enzyme. In this pathway L-arginine is converted to succinate via 2-ketoarginine, 4-guanidinobutyrate, and 4-aminobutyrate with a concomitant production of ammonium, carbon dioxide, and urea (Fig. 2). Ten out of 24 cyanobacterial species have one or two gene(s) encoding an L-arginine oxidase/dehydrogenase (Tables 4 and 9), which is similar to an L-amino acid oxidase that is present in the two closely related strains *Synechococcus elongatus *PCC 6301 and PCC 7942 [22-24]. The corresponding L-amino acid oxidase of these two cyanobacteria is encoded by the *aoxA *genes YP_171306 and ZP_00164087 for *Synechococcus elongatus *PCC 6301 and PCC 7942, respectively, and has been purified and partially characterized. This AoxA has a high specificity for basic L-amino acids as substrate with a preference for L-arginine. AoxA converts L-arginine to 2-ketoarginine and ammonium and utilizes oxygen as electron acceptor. When hydrogen peroxide is not removed by hydrogen peroxide decomposing enzymes, 2-ketoarginine is converted to 4-guanidinobutyrate in a non-enzymatic reaction. Seven of the 10 cyanobacteria, which have a putative L-arginine oxidase/dehydrogenase, also have a gene encoding a putative 4-guanidino butyrase (*Synechococcus *sp. CC 9605, *Synechococcus *sp. WH 7805, *Synechococcus *sp. WH 5701, *Trichodesmium erythraeum *IMS 101, *Synechocystis *sp. PCC 6803, *Nostoc *sp. PCC 7120, and *Nostoc punctiforme *PCC 73102), while the enzyme is absent in *Synechococcus elongatus *PCC 6301, *Synechococcus elongatus *PCC 7942, and *Gloeobacter violaceus *PCC 7421. The genes encoding the two enzymes which convert 4-aminobutyrate to succinate (4-aminobutyrate transaminase and succinate semialdehyde dehydrogenase) are present in all investigated cyanobacteria. The fact that 4-aminobutyrate is also an intermediate in the L-arginine decarboxylase pathway and can additionally be formed by decarboxylation of L-glutamate might explain the presence of these two enzymes even in those cyanobacteria that do not have an L-arginine oxidase/dehydrogenase. An L-arginine oxidase/dehydrogenase pathway, converting L-arginine to 4-aminobutyrate, was first described on the basis of detected products for *Streptomyces griseus *[34] and is also present in *Pseudomonas putida *(Trevisan) Migula P2 ATCC 25571. However, the first enzyme has not yet been characterized biochemically [16,35,36].

### L-arginine succinyl transferase pathway

We did not find evidence for the presence of an L-arginine succinyl transferase pathway in the genome sequences of the investigated 24 cyanobacterial strains. This pathway is suggested to be mainly limited to those heterotrophically growing eubacteria that have the ability to use L-arginine as both, a nitrogen and a carbon source [13,14,16].

### Problems related to the bioinformatic analysis

All 24 investigated cyanobacterial genomes have a putative L-arginine decarboxylase pathway and one or several additional L-arginine-degrading pathways. These can either be an arginase pathway, an L-arginine amidinotransferase pathway, an L-arginine deiminase or an L-arginine oxidase/dehydrogenase pathway. Thus, all investigated cyanobacteria have at least two putative L-arginine-degrading pathways. However, the performed similarity searches do not always allow a statement whether all enzymes of the corresponding pathways are present and whether the gene products have indeed the enzymatic activity that has been assigned to them on the basis of the corresponding similarity searches and domain predictions. No matter what similarity search results suggest, a proof is only provided by activity measurements with purified enzymes. Therefore, uncertainties related to this aspect will be briefly discussed with respect to the enzymes being annotated as ureohydrolases [37] and enzymes being annotated as L-arginine amidinotransferases or L-arginine deiminases. The latter two types of enzymes belong to the family of guanidino group modifiers [38].

#### Ureohydrolases

**Figure 5:**
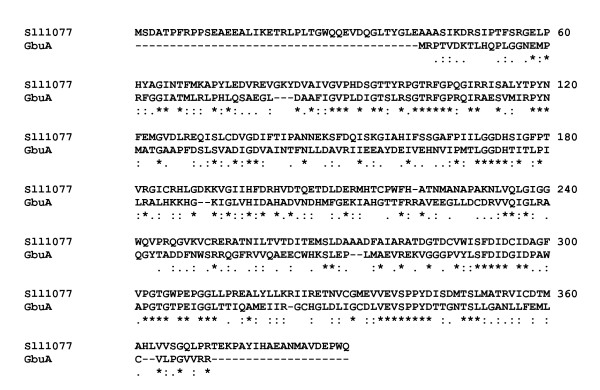
**ClustalW alignment of the putative 4-guanidino butyrase Sll1077 of *Synechocystis *sp. PCC 6803 and the 4-guanidino butyrase GbuA from *Pseudomonas putida *F1 (GbuA_Pputi, ZP_00902038; 25% identical, 20% similar, and 15% weakly similar amino acid residues)**. * identical amino acid residues, : similar amino acid residues (A/V/F/P/M/I/L/W, D/E, R/H/K, S/T/Y/H/C/N/G/Q, and • weakly similar amino acid residues. Gaps were introduced into the sequences to maintain an optimal alignment.

The bioinformatic evaluation of the 24 cyanobacterial genome sequences suggests the presence of (a) gene(s) encoding an arginase, an agmatinase, or a 4-guanidino butyrase in 19 cyanobacterial genomes. Five cyanobacterial species have neither an arginase- nor an agmatinase- nor a 4-guanidino butyrase-encoding gene (Tables 4 and 11). Arginases, agmatinases, and 4-guanidino butyrases release urea from L-arginine (guanidino amino acid), agmatine (guanidino amine) or 4-guanidino butyrate (guanidino acid), respectively. All three types of enzymes belong to the group of ureohydrolases (C-N hydrolases), require the cofactor manganese, and might have an identical evolutionary origin. This implies that an ancient enzyme with broad substrate specificity has progressively been evolved to gain narrower substrate specificity during evolution. Therefore, it is extremely difficult to annotate these genes correctly with respect to the nature of their true substrate [37,39]. According to Sekowska et al. [37], we constructed a phylogenetic distance tree (Fig. 4) with 20 sequences of arginases or agmatinases (given in that paper) as well as the sequences of two arginases from *Arabidopsis thaliana *and the sequences of cyanobacterial ureohydrolases (Table 11). The eukaryotic non-plant arginases cluster in one group (marked in red), while the majority of the cyanobacterial enzymes form two clusters containing either the enzymes from marine cyanobacteria (marked in yellow) or from freshwater cyanobacteria (marked in blue). The two plant arginases form a separate group [28] and are more closely related to agmatinases (encoded by *speB*) than to the arginases from non-photosynthetic organisms of the red cluster. The green cluster contains 4-guanidino butyrases from *Pseudomonas aeruginosa *and *Pseudomonas putida *(GbuA_Paeru and GbuA_Pputi) and the cyanobacterial enzyme Sll1077 of *Synechocystis *sp. PCC 6803 (for relevance of this finding see below) as well as the enzymes of *Synechococcus *sp. CC 9605, *Synechococcus *sp. WH 8102, and *Synechococcus *sp. WH 5701. The similarity of these cyanobacterial enzymes to known 4-guanidino butyrases [40] suggests that these enzymes also have a 4-guanidino butyrase activity (Fig. 4). Since all other cyanobacterial ureohydrolases group into two separate clusters (blue and yellow cluster), it is likely that they do not represent 4-guanidino butyrases, but represent either an arginase or an agmatinase or an enzyme with both activities – albeit with different substrate affinities. It has been shown that the two arginases of *Lycopersicon esculentum *(tomato), which have an arginase activity, also have a very low agmatinase activity (0.2–0.5% of the arginase activity) [28]. Since the blue cluster contains *sll0228 *of *Synechocystis *sp. PCC 6803, which has been shown to encode an agmatinase [21,37], it is likely that at least some of the enzymes in the blue cluster are true agmatinases. To further investigate the real activity of the putative cyanobacterial ureohydrolases, the expression of the corresponding proteins in *E. coli *is required to allow activity measurements as was done for Sll0228 and Sll1077 of *Synechocystis *sp. PCC 6803. Although originally being annotated as arginases, neither Sll0228 nor Sll1077 have arginase activity [21,37]. Sll0228 has been shown to have agmatinase activity, while Sll1077 has neither arginase nor an agmatinase activity [37] and thus, most likely is a 4-guanidino butyrase (alignment of Sll1077 and GbuA from *Pseudomonas putida *F1, ZP_00902038 is given in Fig. 5).

**Table 11:** Genes encoding ureohydrolases in the investigated cyanobacterial marine and freshwater cyanobacteria.

**Strain**	Database entry*	**AA**	**MM (kDa)**	**pI**
**Marine species**				

*Prochlorococcus marinus *SS 120	Pro1849	303	33.6	6.32
*Prochlorococcus marinus *str. MIT 9211	P9211_09067	296	32.7	6.45
*Prochlorococcus marinus *MIT 9312	PMT9312_1779	293	32.6	5.38
*Prochlorococcus marinus *MIT 9313	PMT2214	304	32.8	5.55
*Prochlorococcus marinus *MED 4	PMM1686	294	32.6	5.13
*Prochlorococcus marinus *NATL 2A	PMN2A_1287	299	32.9	5.01
*Synechococcus *sp. CC 9605	Syncc9605_1082 Syncc9605_2591	396 291	43.8 31.3	5.03 4.91
*Synechococcus *sp. CC 9902	Syncc9902_2230	287	30.8	5.10
*Synechococcus *sp. WH 8102	SYNW1412 SYNW2422	426 286	46.8 30.4	5.48 4.68
*Synechococcus *sp. WH 7805	WH7805_06086 WH7805_09974	492 294	53.8 31.5	4.48 4.96
*Synechococcus *sp. WH 5701	WH5701_03860 WH5701_03684	401 308	44.1 32.6	5.35 4.96
*Synechococcus *sp. RS 9917	RS9917_06190	286	30.9	5.06
*Crocosphaera watsonii *WH 8501	n.d.	n.d.	n.d.	n.d.
*Trichodesmium erythraeum *IMS 101	Tery_3780	303	34.0	4.80

**Freshwater species**				

*Synechococcus elongatus *sp. PCC 6301	n.d.	n.d.	n.d.	n.d.
*Synechococcus elongatus *sp. PCC 7942	n.d.	n.d.	n.d.	n.d.
*Synechococcus Yellowstone *sp. JA-3-3-AB	CYA_0859	301	33.1	5.51
*Synechococcus Yellowstone *sp. JA-2-3Bα (2–13)	CYB_1744	307	33.7	5.23
*Thermosynechococcus elongatus *BP-1	n.d.	n.d.	n.d.	n.d.
*Synechocystis *sp. PCC 6803	Sll1077 Sll0228	390 306	42.9 33.5	5.06 4.90
*Gloeobacter violaceus *PCC 7421	n.d.	n.d.	n.d.	n.d.
*Nostoc *sp. PCC 7120	Alr2310	346	38.6	4.69
*Nostoc punctiforme *PCC 73102	Npun02002114	347	38.5	4.53
*Anabaena variabilis *ATCC 29413	Ava_0127	346	38.5	4.66

N.d. = not detected. *These ureohydrolases are annotated as arginases, as agmatinases as well as 4-guanidino butyrases. The (+) in Table 3 for A2.1, B1, and E2 refers to an identical gene, because the gene annotation does not distinguish between arginases, agmatinases, and 4-guanidino butyrases. A classification is only possible in a few cases, in which enzymatic activity has been measured or the similarity values are very high to already biochemically well-characterized enzymes (see text for details).

#### Enzymes modifying the guanidino group

This family of enzymes comprises L-arginine deiminases and L-arginine amidinotransferases [38,41], which share common structural features [41]. L-arginine deiminases participate in L-arginine catabolism and are found in prokaryotes [13,16,42] and primitive eukaryotes [30]. L-arginine amidinotransferases have been shown to have a function as L-arginine:glycine amidinotransferase in creatine biosynthesis in vertebrates [43,44], as L-arginine:glycine amidinotransferase in the biosynthesis of the toxin cylindrospermopsin in various cyanobacteria [45], as L-arginine:inosamine phosphate amidinotransferase in streptomycin biosynthesis in *Streptomyces *spp. [45], and as L-arginine:L-lysine amidinotransferase in the phaseolotoxin biosynthesis in *Pseudomonas syringae *pv. *phaseolicola *[46]. In nine cyanobacteria an identical gene was annotated as L-arginine amidinotransferase as well as L-arginine deiminase (Table 4). Thus, a decision, which of the two putative pathways is present, can not be made with certainty. The similarity of the cyanobacterial enzymes to characterized L-arginine deiminases is rather low and is even lower to L-arginine amidinotransferases (Table 12). However, since L-arginine amidinotransferases have so far only been shown to function in antibiotic or toxin biosynthesis in prokaryotes and since an L-arginine deiminase activity has been detected in several fresh water cyanobacteria [17-20], we think that it is more likely that the corresponding gene in the nine cyanobacteria (Tables 4, 7, and 8) encodes an L-arginine deiminase and not an L-arginine amidinotransferase. One reason, why these genes have not yet been annotated as L-arginine deiminases in the databases, may be related to the fact that so far well characterized prokaryotic L-arginine deiminases consist of about 400 amino acid residues (Table 12) [47-49] and that the L-arginine deiminase of the primitive eukaryote *Giardia intestinalis *consists of 580 amino acid residues [30]. In contrast, the corresponding nine cyanobacterial genes encode proteins of 699 to 710 amino acid residues length with a molecular mass of 77.5 to 78.3 kDa. Among the cyanobacterial proteins a high similarity of about 80% exists (Table 12). Another unique property of cyanobacterial L-arginine deiminases is that they contain two transmembrane helixes in their C-terminal region. This implies that the cyanobacterial enzymes are membrane-bound or at least membrane-associated. Whether the enzymes are bound to the cytoplasmic or the thylakoid membrane is not yet known.

**Table 12:** Comparison of cyanobacterial putative L-arginine deiminases or L-arginine amidinotransferases to selected prokaryotic sequences and a sequence of a primitive eukaryote*.

**Strain**	**Database entry**	**AA**	**MM (kDa)**	**pI**	**Identity/similarity/gaps vs. Sll1336 (%)**
**Cyanobacterial L-arginine deiminases or L-arginine amidinotransferases**					

*Synechocystis *sp. PCC 6803	Sll1336	705	78.3	5.40	100.0/100.0/0.0

*Crocosphaera watsonii *WH 8501	CwatDRAFT_0830	703	78.0	5.15	78.0/88.8/0.3
*Trichodesmium erythraeum *IMS 101	Tery_4659	703	77.8	5.43	74.3/85.7/1.1
*Synechococcus Yellowstone *sp. JA-2-3Bα (2–13)	YP_476511	710	78.2	5.75	64.1/79.0/2.1
*Thermosynechococcus elongatus *BP-1	Tll0507	699	77.5	5.53	71.3/84.9/1.4
*Gloeobacter violaceus *PCC 7421	Glr1758	699	77.5	5.53	63.7/78.6/2.1
*Nostoc *sp. PCC 7120	Alr4995	703	77.9	5.41	73.4/85.7/0.8
*Nostoc punctiforme *PCC 73102	Npun02001803	703	77.9	5.48	74.6/86.6/1.4
*Anabaena variabilis *ATCC 29413	Ava_2273	703	78.2	5.38	73.7/86.6/0.8

L-arginine deiminases of prokaryotes and a primitive eukaryote*					

Giardia intestinales*	AAC06116	580	64.1	6.11	13.9/22.3/53.1
*Thermoplasma volcanium *GSS1	NP_110996	418	48.1	5.32	10.2/18.1/65.7
*Thermoplasma acidophilum *DSM 1728	NP_394447	418	47.7	5.20	8.7/17.5/65.5
*Pseudomonas aeruginosa*	P13981	418	46.4	5.52	7.3/12.0/74.9
*Enterococcus faecalis*	CAC41341	408	46.7	4.87	7.4/14.8/71.6
*Bacillus licheniformis*	AAU25597	411	47.2	5.28	7.8/13.2/73.3

**Characterized L-arginine amidinotransferases**					

*Rattus norvegicus*	AAA21250	423	48.2	7.17	6.3/9.5/82.1
*Streptomyces griseus*	CAA68517	347	38.7	5.12	9.0/12.7/72.5
*Aphanizoemon ovalisporum*	AAM33469	392	44.8	5.40	8.0/13.2/74.3

L-arginine deiminases and L-arginine amidinotransferases belong to a superfamily of enzymes that catalyze the modification of guanidino groups. The number of amino acid residues, the molecular mass, and the calculated isoelectric point is given. Moreover, the similarity of the selected reference enzymes to Sll1336 from *Synechocystis *sp. PCC 6803 is given. Values for % identity and similarity to Sll1336 were determined with the EMBOSS Pairwise alignment algorithm [65]. The percentage identity and similarity does not include weakly similar amino acid residues.

### Identification of genes encoding enzymes of L-arginine catabolizing pathways in Synechocystis sp. PCC 6803

We chose *Synechocystis *sp. PCC 6803 as a model organism to present more details on the enzymes of the L-arginine-degrading pathways and to validate the bioinformatic results by a transcript analysis. The reason for choosing this cyanobacterium is based on previously published results, showing that *Synechocystis *sp. PCC 6803 possesses a very effective uptake system for L-arginine [50]. Moreover, several products of L-arginine degradation have already been identified [51]. In addition, substantial differences in the utilization of L-arginine as sole N-source in the growth medium have been observed between *Synechocystis *sp. PCC 6803 WT and a PsbO-free *Synechocystis *mutant [10].

**Figure 6:**
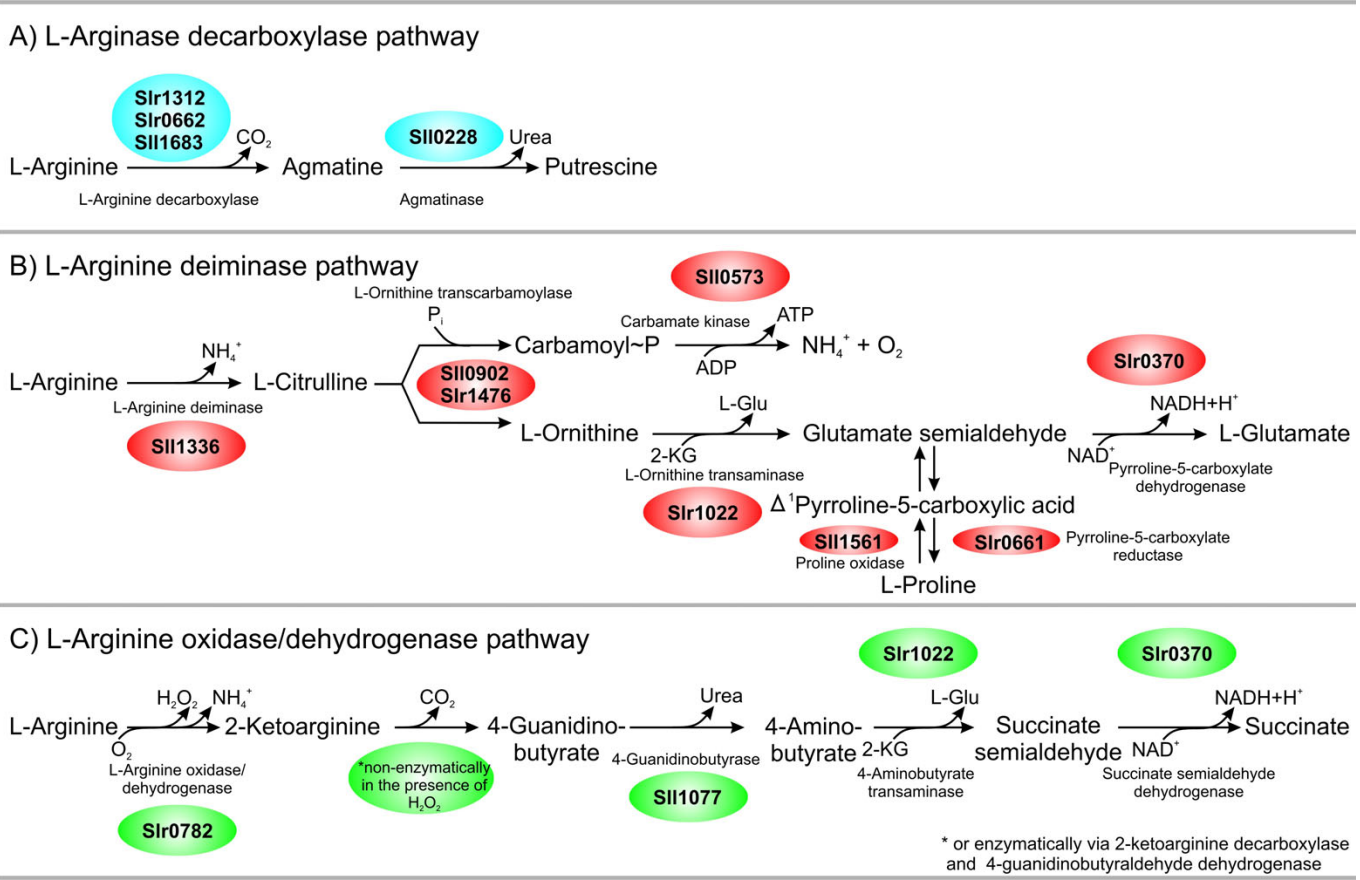
**Schematic presentation of the three L-arginine-degrading pathways in *Synechocystis *sp. PCC 6803 with the corresponding enzymes, intermediates, cofactors, and final products**. **A)**. L-arginine decarboxylase pathway most likely only provides polyamines and ammonia. **B) **L-arginine deiminase pathway degrades L-arginine via L-citrulline to L-ornithine and carbamoyl phosphate. L-ornithine is further metabolized via glutamate semialdehyde to L-glutamate. Glutamate semialdehyde can also be converted to L-proline via Δ^1^pyrroline-5-carboxylate. Carbamoyl phosphate is further metabolized to ammonium and carbon dioxide. This enzymatic reaction is catalyzed by the enzyme carbamate kinase and is coupled to ATP synthesis. **C) **The L-arginine oxidase/dehydrogenase pathway converts L-arginine to succinate via 2-ketoarginine, 4-guanidinobutyrate, 4-aminobutyrate, and succinate semialdehyde.

*Synechocystis *sp. PCC 6803 contains genes encoding enzymes of a putative L-arginine decarboxylase pathway, an L-arginine deiminase pathway, and an L-arginine oxidase/dehydrogenase pathway (Tables 3, 4, 13, and Fig. 6).

**Table 13:** Presence of genes in the *Synechocystis *sp. PCC 6803 genome encoding putative enzymes of an L-arginine decarboxylase-, an L-arginine deiminase-, and an L-arginine oxidase/dehydrogenase pathway.

**L-arginine-degrading pathways in *Synechocystis *sp. PCC 6803**	**ORF**	**Database #**	**Length (aa)**	**pI**	**MW (kDa)**	**Best hit vs. gene**	**Organism**	**E-value**	**Similarity (ident./pos. aa)**
**L-Arginine decarboxylase**									

L-Arginine decarboxylase (A1)	*sll1683*	NP_440109	483	5.44	51.84	*speA*	*B. subtilis*	5.0e-103	40/61
	*slr0662*	NP_442871	695	5.08	78.24	*speA*	*X. campestris*	2.0e-134	41/56
	*slr1312*	NP_439907	659	5.30	74.48	*speA*	*X. campestris*	5.0e-121	38/56
Agmatinase (A2.1)	*sll1077*	NP_440618	390	5.06	42.96	*speB2*	*P. aeruginosa*	1.1e-40	33/41
	*sll0228*	NP_440030	306	4.90	33.46	*speB*	*B. subtilis*	1.6e-22	30/45
Putrescine oxidase or transaminase (A3)	n.d.	n.d.	n.d.	n.d.	n.d.	n.d.	n.d.	n.d.	n.d.
4-Aminobutyraldehyde dehydrogenase (A4)	*sll1495*	NP_442886	397	8.43	43.54	*BMEII0291*	*B. melitensis*	1.2e-93	42/61
4-Aminobutyrate transaminase (A5)	*slr1022*	NP_440479	429	5.11	46.54	*gabT*	*P. aeruginosa*	6.7e-58	33/50
	*sll0017*	NP_442115	433	5.13	45.87	*gabT*	*E. coli*	5.7e-41	30/44
Succinate semialdehyde dehydrogenase (A6)	*slr0370*	NP_442020	454	5.02	48.75	*gabD*	*X. campestris*	5.0e-121	47/65
	*sll1561*	NP_441689	990	5.46	110.03	*gabD*	*P. aeruginosa*	2.7e-66	17/25

**L-Arginine deiminase**									

L-Arginine deiminase (D1)	*sll1336*	NP_442829	705	5.40	78.33	*cyb_250*	*S. yellowstone*	0.0	61/79
L-Ornithine transcarbamoylase (D2)	*sll0902* * slr1476*	NP_442776 NP_441572	308 331	5.38 6.53	33.62 33.39	*argF* * argF*	*P. aeruginosa* * P. aeruginosa*	1.1e-77 7.2e-13	47/66 26/42
Carbamate kinase (D3)	*sll0573*	NP_443041	308	5.66	32.93	*ygcA*	*E. coli*	8.1e-52	41/58
L-Ornithine transaminase (D4)	*slr1022*	NP_440479	429	5.11	46.54	*rocD*	*B. subtilis*	2.1e-61	32/52
Δ^1^Pyrroline-5-carboxylate dehydrogenase (D5)	*slr0370*	NP_442020	454	5.02	48.75	*ycgN*	*B. subtilis*	7.9e-40	26/40
Δ^1^Pyrroline-5-carboxylate reductase	*slr0661*	NP_442689	128	5.11	14.4	*slr0661*	*S. PCC *6803	0.0	100/100
Proline oxidase	*sll1561*	NP_441689	990	5.46	110.03	*rocA*	*B. subtilis*	6.0e-138	25/34

**L-Arginine oxidase/dehydrogenase**									

L-Arginine oxidase/dehydrogenase (E1)	*slr0782*	NP_442072	471	5.19	51.37	*aoxA*	*S. elongatus*	1.7e-18	20/35
4-Guanidino butyrase (E2)	*sll1077*	NP_440618	390	5.06	42.96	*gbuA*	*P. aeruginosa*	1.1e-40	26/41
	*sll0228*	NP_440030	306	4.90	33.46	*gbuA*	*P. aeruginosa*	1.1e-19	26/41
4-Aminobutyrate transaminase (E3)	*slr1022*	NP_440479	429	5.11	46.54	*gabT*	*P. aeruginosa*	6.7e-58	33/50
	*sll0017*	NP_442115	433	5.13	45.87	*gabT*	*P. aeruginosa*	5.7e-41	30/44
Succinate semialdehyde dehydrogenase (E4)	*slr0370*	NP_442020	454	5.02	48.75	*gabD*	*X. campestris*	5.0e-121	47/65
	*sll1561*	NP_441689	990	5.46	110.03	*gabD*	*P. aeruginosa*	2.7e-66	17/25

The letters with numbers in parenthesis behind the enzyme names correspond to those given in Tables 3 and 4, and Fig. 2. In *Synechocystis *sp. PCC 6803 the gene *slr1022 *has similarity to L-ornithine transaminases and to 4-aminobutyrate transaminases. The L-ornithine transferase (D2) and the 4-aminobutyrate transferase (E3) both belong to the group of class III aminotransferases (InterProScan), which explains why the same gene *slr1022 *is annotated either as L-ornithine transaminase or as 4-aminobutyrate transaminase. The gene *slr0370 *has similarity to the Δ^1^pyrroline-5-carboxylate dehydrogenase (D5) and to succinate semialdehyde dehydrogenase (E4). Both enzymes belong to the NAD-dependent aldehyde dehydrogenases (InterProScan), which explains why the same gene *slr0370 *is either annotated as Δ^1^pyrroline-5-carboxylate dehydrogenase or succinate semialdehyde dehydrogenase Thus, it can not be decided in a bioinformatic approach whether the gene products Slr1022 and Slr0370 are components of the L-arginine deiminase pathway or the L-arginine oxidase/dehydrogenase pathway or of both pathways. N.d. = not detected.

Three genes, *sll1683*, *slr0662*, and *slr1312*, encoding enzymes with similarity to L-arginine decarboxylases, are present. As shown in Table 10, Sll1683 has a higher similarity to the biodegradable than to the biosynthetic L-arginine decarboxylase of *E. coli*. In contrast, Slr0662 and Slr1312 have higher similarity to the biosynthetic than to the biodegradable enzyme. Moreover, two genes, *sll1077 *and *sll0228*, encoding proteins with similarity to ureohydrolases, were detected. Sll0228, but not Sll1077, has been shown to have agmatinase activity, catalyzing the synthesis of putrescine [21,37]. However, no true putrescine oxidase or putrescine transaminase encoding genes were found in the genome of *Synechocystis *sp. PCC 6803. Therefore, the L-arginine decarboxylase pathway may mainly serve as a route for polyamine biosynthesis and for the production of ammonium from L-arginine. This assumption is in agreement with results obtained for pseudomonads, which were shown to an L-arginine decarboxylase pathway [13,14,16].

**Figure 7:**
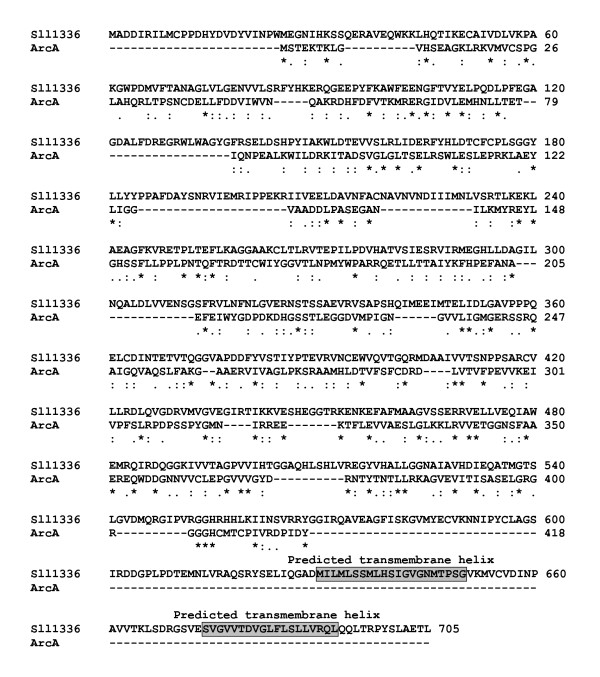
**ClustalW alignment of the putative L-arginine deiminase Sll1336 of *Synechocystis *sp. PCC 6803 and the L-arginine deiminase ArcA from the primitive eukaryote *Giardia intestinales***. Both proteins share 43% overall similarity (10% identical, 19% strongly similar, 14% weakly similar amino acid residues. * Identical amino acid residues, : similar amino acid residues (A/V/F/P/M/I/L/W, D/E, R/H/K, S/T/Y/H/C/N/G/Q, and • weakly similar amino acid residues. Gaps were introduced into the sequences to maintain an optimal alignment. Two putative transmembrane helices of Sll0573 are boxed (see text for details).

Sll1336 has the common features of an L-arginine amidinotransferase as well as of an L-arginine deiminase. However, since L-arginine amidinotransferases are predominantly involved in antibiotic or toxin synthesis in prokaryotes, it is more likely that Sll1336 is an L-arginine deiminase. This is supported by the fact that Sll1336 has a slightly higher similarity to sequenced L-arginine deiminases than to L-arginine amidinotransferases (Table 12). The highest similarity of Sll1336 (705 aa) exists to the L-arginine deiminase ArcA from *Giardia intestinales *(580 aa, 43% overall similar amino acid residues: 10% identical, 19% strongly similar, and 14% weakly similar amino acid residues). Thus, Sll1336 (705 aa) is substantially larger than the average L-arginine deiminases of primitive eukaryotes (~580 aa) or of heterotrophically growing prokaryotes (~400 aa) (Table 12 and Fig. 7). In contrast to the bacterial enzymes, the L-arginine deiminase of *Synechocystis *sp. PCC 6803 (and of all other investigated cyanobacterial species) also has two putative transmembrane helices in the C-terminal region between the amino acid residues 630 to 651 and between the amino acid residues 674 and 692 (Fig. 7). The prediction was carried out with three different software packages (DAS Transmembrane Prediction Server [52]; TMpred Server [53]; TopPred Server [54]. Therefore, Sll1336 is bound either to the cytoplasmic or the thylakoid membrane.

**Figure 8:**
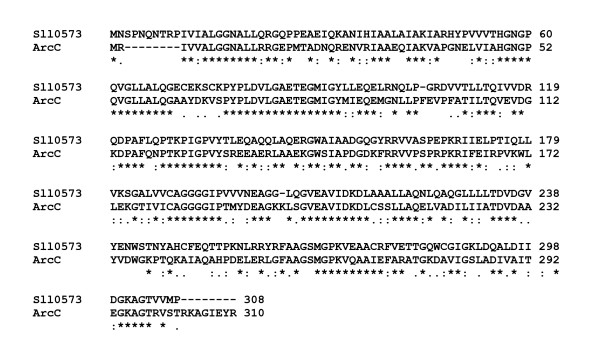
**ClustalW alignment of the putative carbamate kinase Sll0573 of *Synechocystis *sp. PCC 6803 and the carbamate kinase ArcC from *Pseudomonas aeruginosa***. Both proteins share 82% overall similarity (55% identical, 18% strongly similar, 9% weakly similar amino acid residues. * Identical amino acid residues, : similar amino acid residues (A/V/F/P/M/I/L/W, D/E, R/H/K, S/T/Y/H/C/N/G/Q, and • weakly similar amino acid residues. Gaps were introduced into the sequences to maintain an optimal alignment. Two putative transmembrane helices of Sll0573 are boxed (see text for details).

Like all other investigated cyanobacteria, *Synechocystis *sp. PCC 6803 has an L-ornithine transcarbamoylase (Slr1022), but it is the only species among the investigated strains, which has a gene encoding a carbamate kinase (*sll0573*). This enzyme shows an intriguingly high degree of similarity to carbamate kinases from other eubacteria. Sll0573 (32 kDa and calculated pI 5.66) has an overall similarity of 71% (41% identical, 19% strongly similar, and 11% weakly similar amino acid residues) to the carbamate kinase ArcC from *Enterococcus faecalis *(32.9 kDa and calculated pI 5.13) and an overall similarity of 82% (55% identical, 18% strongly similar, 9% weakly similar amino acid residues) to ArcC from *Pseudomonas aeruginosa *(33 kDa and calculated pI 5.25) (Fig. 8). Thus, it is likely that the second possible route for L-arginine degradation in *Synechocystis *sp. PCC 6803 is an L-arginine deiminase pathway leading to synthesis of L-citrulline and subsequently to L-ornithine, carbon dioxide, ammonium, and ATP (Fig. 6). L-ornithine becomes further metabolized to L-glutamate by an L-ornithine transaminase (Slr1022) and a Δ^1^pyrroline-5-carboxylate dehydrogenase (Slr0370) (Table 11). This pathway also leads to the synthesis of L-proline via a Δ^1^pyrroline-5-carboxylate reductase (ProC, Slr0661), and L-proline can be converted back to this intermediate by a proline oxidase (PutA, Sll1561) [21].

The third possible route of L-arginine catabolism in *Synechocystis *sp. PCC 6803 may be an L-arginine oxidase/dehydrogenase pathway. The gene *slr0782 *encodes a putative L-arginine oxidase/dehydrogenase, *sll1077 *and *sll0228 *encode putative ureohydrolases, *slr1022 *and *sll0017 *encode putative 4-aminobutyrate transaminases, and *slr0370*, *sll1561*, and *slr0091 *encode putative succinate semialdehyde dehydrogenases. Thus, L-arginine becomes degraded to succinate, carbon dioxide, and ammonium, via 2-ketoarginine, 4-guanidinobutyrate, and 4-aminobutyrate. Since the ureohydrolase Sll1077 groups with known 4-guanidino butyrases (Fig. 4), and the heterologously expressed enzyme has neither an arginase nor an agmatinase activity [37], this enzyme may indeed be a 4-guanidino butyrase. An alignment of the enzyme with the biochemically identified 4-guanidino butyrase of *Pseudomonas putida *strain F1 (ZP_00902038) is given (Fig. 5).

**Figure 9:**
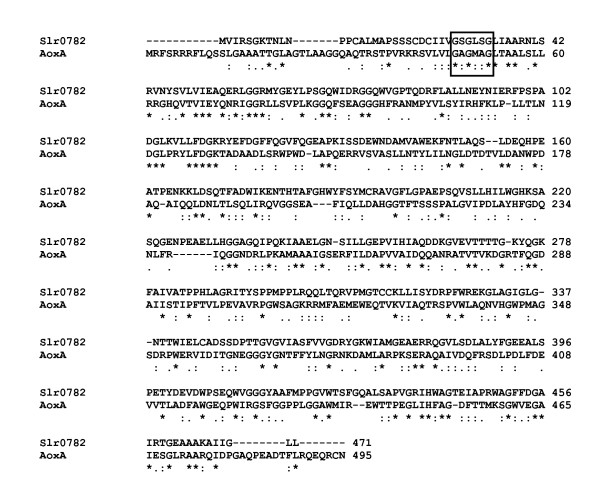
**ClustalW alignment of the putative L-arginine oxidase/dehydrogenase Slr0782 from *Synechocystis *sp. PCC 6803 with the characterized L-amino acid oxidase AoxA from *Synechococcus elongatus *PCC 6301 (P72346) [23]**. Both proteins share an overall similarity of 57% (21% identical, 23% similar, and 13% weakly amino acid residues). The dinucleotide binding motif GxGxxG is boxed. * Identical amino acid residues, : similar amino acid residues (A/V/F/P/M/I/L/W, D/E, R/H/K, S/T/Y/H/C/N/G/Q, and • weakly similar amino acid residues. Gaps were introduced into the sequences to maintain an optimal alignment. Two putative transmembrane helices (aa 628–648; aa 670–690) were detected for Slr0782 using the DAS TM prediction algorithm [52]. Slr0782 also has 66% similarity (31% identical; 22% strongly similar, and 13% weakly similar amino acid residues) to AoxB of *Synechococcus elongatus *PCC 6301, an enzyme not yet characterized.

The first enzyme of the L-arginine oxidase/dehydrogenase pathway (Slr0782) in *Synechocystis *sp. PCC 6803 has 58% similarity (20% identical, 24% similar, and 14% weakly similar amino acid residues) to an L-amino acid oxidase (AoxA) from *Synechococcus elongatus *PCC 6301, encoded by the *aoxA *gene (YP_171306) [22-24]. This enzyme catalyzes the oxidative deamination of basic L-amino acids with a preference for L-arginine. An alignment of Slr0782 with AoxA of *Synechococcus elongatus *PCC 6301 is given and shows that Slr0782 has a dinucleotide-binding site (GxGxxG) [55] like the AoxA enzyme (Fig. 9). Thus, Slr0782 may also be a FAD-containing enzyme. Since we were never able to detect an L-arginine oxidizing activity with utilization of molecular oxygen in intact cells or cell extracts of *Synechocystis *sp. PCC 6803 so far (unpublished results), it is more likely that Slr0782 interacts in a complex not yet understood way with the electron transport chain. This is in agreement with the fact that the enzyme has two hydrophobic regions possibly being transmembrane helices. We would like to also point out that *Synechococcus elongatus *PCC 6301 has an additional gene encoding a protein called AoxB (YP_171854), which has 59% similarity (25% identical, 21% similar, and 13% weakly similar amino acid residues) to AoxA [24]. AoxB has not yet been characterized biochemically. Slr0782 of *Synechocystis *sp. PCC 6803 has a higher similarity to AoxB (in total 66% similarity: 31% identical, 22% similar, and 13% weakly similar amino acid residues) than to AoxA (in total 58% similarity). It should also be mentioned that the genomes of different *Pseudomonas *species contain a gene encoding an enzyme, which has similarity to Slr0782 (*P. putida *KT2440, NP_747085; *P. putida *F1, ZP_00902633; *P. aeruginosa *PAO-1, NP_249112; *P fluorescens *PfO-1, YP_348469). The similarity of Slr0782 to the enzyme of *P. fluorescens *corresponds to 47% (27% identical, 17% similar, and 13% weakly similar amino acid residues). All these enzymes contain a dinucleotide-binding GxGxxG motif and thus, are likely FAD-containing dehydrogenases and not aminotransferases [35,36]. For *Pseudomonas putida *(Trevisan) Migula P2 ATCC 2557 the Rodwell group has indeed suggested that an L-amino acid oxidase is the first enzyme degrading L-arginine via 2-ketoarginine, 4-guanidinobutyrate, and 4-aminobutyrate to succinate [35,36].

### Detection of transcripts for L-arginine-degrading enzymes in *Synechocystis *sp. PCC 6803

The bioinformatic evaluation suggests the presence of three putative L-arginine-degrading pathways in *Synechocystis *sp. PCC 6803. These putative pathways are an L-arginine decarboxylase pathway (three isoenzymes as first enzyme: Sll1683, Slr0662, and Slr1312), an L-arginine deiminase pathway (first enzyme Sll1336), and an L-arginine oxidase/dehydrogenase pathway (first enzyme Slr0782) (Fig. 6).

**Figure 10:**
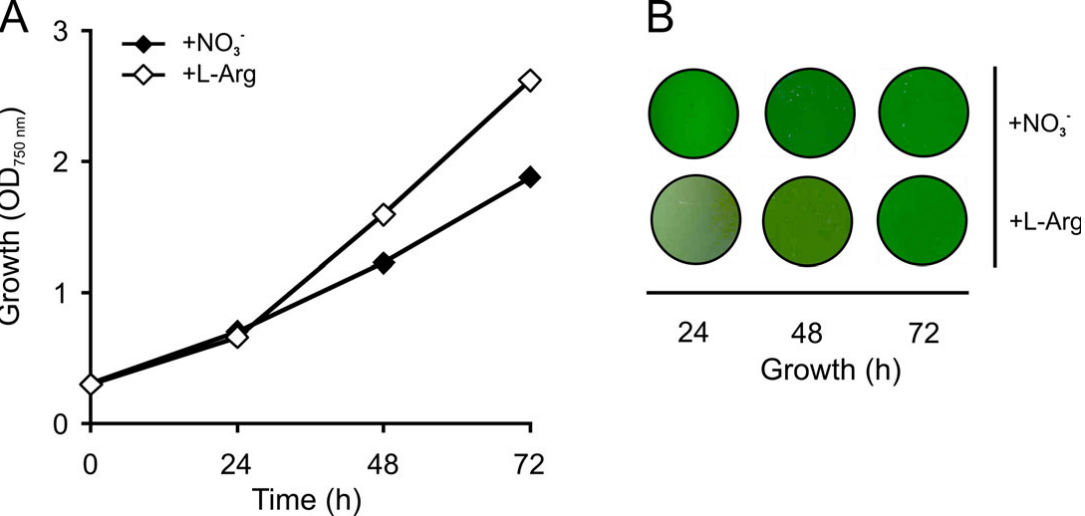
Growth and phenotypical appearance of *Synechocystis *sp. PCC 6803 cells grown in the presence of nitrate or L-arginine as sole N-source and with a light intensity of 50 μmol photons m^-2 ^s^-1 ^for 24, 48 or 72 hours.

**Figure 11:**
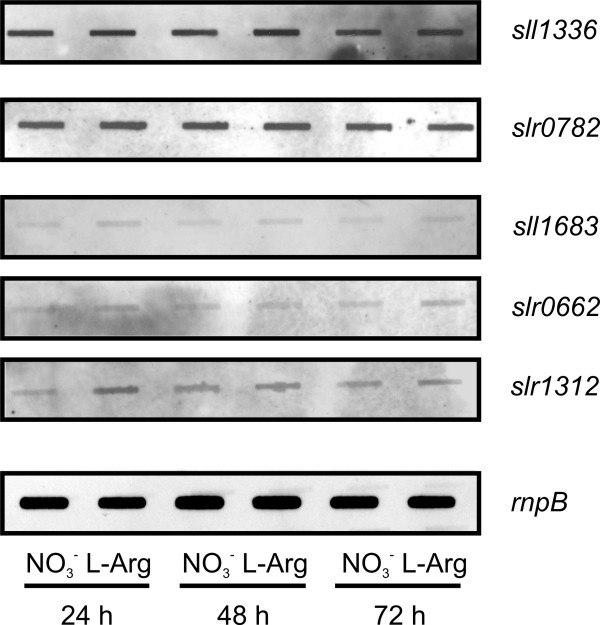
**Slot-blot transcript analysis of the genes encoding the first putative enzymes of the L-arginine deiminase pathway (*sll1336*), the L-arginine oxidase/dehydrogenase pathway (*slr0782*), and the L-arginine decarboxylase pathway (three possible deiminase-encoding genes: *sll1683*, *sll0662*, and *slr1312*) in *Synechocystis *sp. PCC 6803**. *Synechocystis *sp. PCC 6803 cells were grown for 24, 48, or 72 h with nitrate or L-arginine as sole N-source and with a constant illumination of 50 μmol photons m^-2 ^s^-1^. Steady state transcript pools were investigated with gene-specific probes of equal length and equal GC % content to assure equal labeling with Dig-dUTP. An *rnpB*-specific probed was used to assure equal loading. The figure allows for a direct comparison of the various transcript concentrations. Moreover, changes in transcript level can be compared in cells grown with L-arginine (increase or decrease) to that grown with nitrate.

For detection of the corresponding transcripts, *Synechocystis *sp. PCC 6803 was cultivated with nitrate or with L-arginine as sole N-source and with an illumination of 50 μmol photons m^-2 ^s^-1 ^for three days. These growth conditions were similar to those published previously [51] for experiments to determine products of L-arginine degradation. The growth curves and the chlorophyll content are given in Fig. 10. *Synechocystis *sp. PCC 6803 grew about equally well with nitrate as with L-arginine. Total RNA was isolated from the corresponding cultures and was applied to RNA slot-blot hybridization with selected Dig-dUTP-labeled gene-specific DNA probes (Fig. 11). Equal length, concentration, almost equal GC-content of the probes, and equal exposure time allowed for semi-quantitative comparison of mRNA levels of all five investigated transcripts: *sll1683*, *sll0662*, and *slr1312 *encoding isoenzymes of L-arginine decarboxylases, *sll1336 *encoding an L-arginine deiminase, and *slr0782 *encoding an L-arginine oxidase/dehydrogenase. The transcript level for the three L-arginine decarboxylase-encoding genes was low when the cells grew with nitrate and did not or only slightly increase when the cells grew with L-arginine as sole N-source. A low steady-state mRNA level was also observed for *sll0228 *transcript (not shown), which encodes an agmatinase-type enzyme [37,51] – the second enzyme in the L-arginine decarboxylase pathway. This implies that the L-arginine decarboxylase pathway probably has its only function in polyamine biosynthesis and does not represent a major pathway for L-arginine degradation in *Synechocystis *sp. PCC 6803 when cells grew with L-arginine as sole N-source.

**Figure 12:**
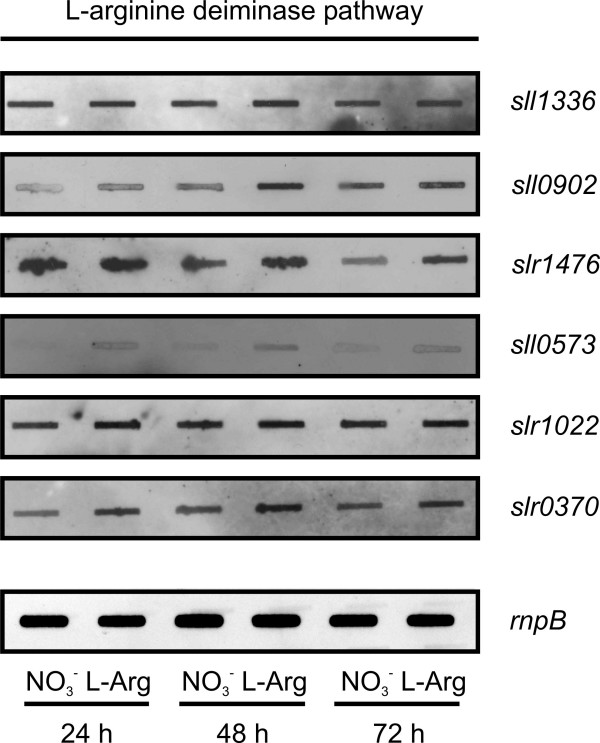
**Slot-blot transcript analysis of the genes encoding the putative enzymes of the L-arginine deiminase pathway in *Synechocystis *sp. PCC 6803**. *Synechocystis *sp. PCC 6803 cells were grown for 24, 48, or 72 h with nitrate or L-arginine as sole N-source and with a constant illumination of 50 photons m^-2 ^s^-1^. Steady state transcript pools were investigated with gene-specific probes of equal length and equal GC % content to assure equal labeling with Dig-dUTP. An *rnpB*-specific probed was used to assure equal loading. The figure allows for the direct comparison of transcript levels between cells grown with L-arginine to that grown with nitrate.

**Figure 13:**
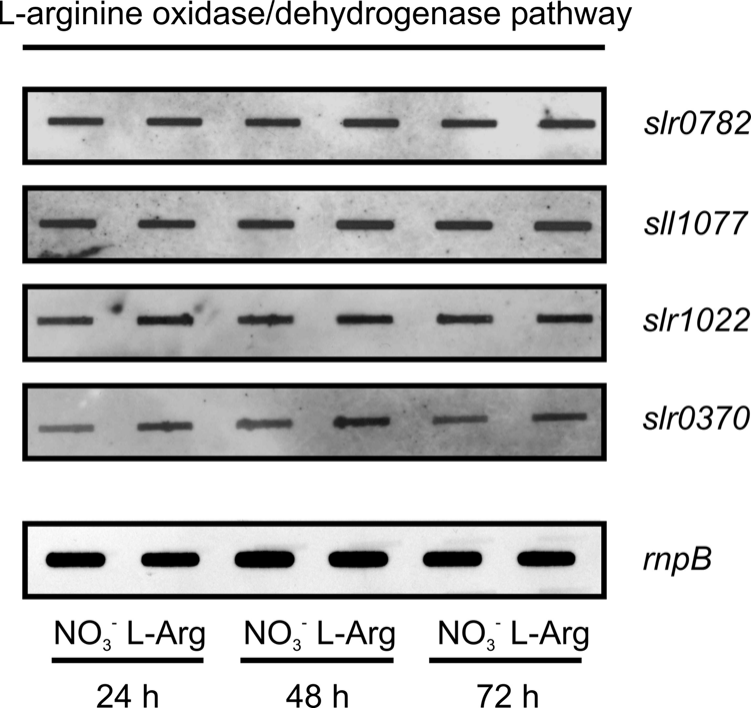
**Slot-blot transcript analysis of the genes encoding the putative enzymes of the L-arginine oxidase/dehydrogenase pathway in *Synechocystis *sp. PCC 6803**. *Synechocystis *sp. PCC 6803 cells were grown for 24, 48, or 72 h with nitrate or L-arginine as sole N-source and with a constant illumination of 50 photons m^-2 ^s^-1^. Steady state transcript pools were investigated with gene-specific probes of equal length and equal GC% content to assure equal labeling with Dig-dUTP. An *rnpB*-specific probed was used to assure equal loading. The figure allows for the direct comparison of transcript levels between cells grown with L-arginine to that grown with nitrate.

As shown in Fig. 11, the transcript levels for the L-arginine deiminase (Sll1336) as well as for the L-arginine oxidase/dehydrogenase (Slr0782) were substantially higher than for the three L-arginine decarboxylase isoenzymes. The steady-state transcript levels for these two enzymes were as high in nitrate-grown cells as in L-arginine-grown cells. This suggests that these two genes are transcribed constitutively. The same is true for the transcripts of the subsequent enzymes of the two pathways with the exception of the carbamate kinase transcript (Fig. 12 and 13). The mRNA for the carbamate kinase was lower than for the other enzymes and the steady-state transcript level was found to be highly increased in L-arginine-grown cells.

## Conclusion

The bioinformatic evaluation of 24 cyanobacterial genomes suggests the presence of an L-arginine decarboxylase-, an arginase-, an L-arginine amidinotransferase-, an L-arginine deiminase-, and an L-arginine oxidase/dehydrogenase pathway in the investigated cyanobacteria (Tables 3 and 4, and Fig. 2). All investigated strains contain an L-arginine decarboxylase pathway, which most likely mainly facilitates polyamine biosynthesis. Since extracellularly added putrescine has been shown to be toxic, at least for some cyanobacteria [56], it is unlikely that this pathway is a major pathway for L-arginine degradation. In addition to the L-arginine decarboxylase pathway, one or two further L-arginine-degrading pathway(s) is (are) present, which is either an arginase pathway, an L-arginine deiminase pathway or an L-arginine oxidase/dehydrogenase pathway. Although an L-arginine amidinotransferase pathway can not be excluded entirely, this pathway is rather unlikely to have a major function in L-arginine degradation, since L-arginine amidinotransferases seem to mainly function in antibiotic and toxin production in prokaryotes [44-46].

An interesting result of the bioinformatic analysis is the observation that the cyanobacterial L-arginine deiminases, being present in nine cyanobacterial strains (Table 4), are substantially larger than the corresponding enzymes from non-photosynthetic eubacteria (Table 12). Further, they seem to be bound either to the cytoplasmic or the thylakoid membrane. In bacteria it has been shown that the L-arginine deiminase pathway is regulated in a rather complex way in dependence of the L-arginine and oxygen concentration, the redox poise, and/or energy status of the cell [13,14,48,49]. On the basis of the larger size and the predicted membrane association of the cyanobacterial L-arginine deiminases, the regulation of the L-arginine deiminase pathway in cyanobacteria maybe even more complex than in bacteria. This has also to be seen under the aspect that this pathway leads to ATP synthesis in the last enzymatic step providing an additional substrate-level phosphorylation site.

The second rather unexpected observation is the presence of a putative L-arginine oxidase/dehydrogenase pathway in ten cyanobacteria (Table 4). The first enzyme of this pathway has similarity to an L-amino acid oxidase, catalyzing the oxidative deamination of basic L-amino acids with a preference for L-arginine and with oxygen as electron acceptor in *Synechococcus elongatus *PCC 6301 and PCC 7942. This pathway has not yet been investigated in detail. However, preliminary results, which had been obtained with *Synechocystis *sp. PCC 6803, suggest that the first enzyme of this pathway does not represent an L-arginine oxidase with oxygen as electron acceptor, but rather represents an L-arginine dehydrogenase, which interacts in a complex not yet understood with the electron transport chain. An interaction of amino acid dehydrogenases with the respiratory electron transport chain has previously been shown for *E. coli *[57].

In addition to the overview on L-arginine-degrading pathways in 24 cyanobacteria, we have performed a more detailed evaluation of the pathways in *Synechocystis *sp. PCC 6803. This investigation provided evidence that *Synechocystis *sp. PCC 6803 has three putative L-arginine-degrading pathways, being an L-arginine decarboxylase pathway, an L-arginine deiminase pathway, and an L-arginine oxidase/dehydrogenase pathway. An arginase pathway does not seem to exist, since the two proteins, originally annotated as arginases, do not possess an arginase activity [37,51]. Transcript analyses revealed that the mRNA levels for the three isoenzymes of L-arginine decarboxylase (Slr1312, Slr0662, and Sll1683) and also for the agmatinase Sll0228 were rather low in *Synechocystis *sp. PCC 6803 in nitrate- or L-arginine-grown cells. Thus, this pathway probably has its major function in polyamine biosynthesis. In contrast, the transcript levels for a putative L-arginine deiminase pathway (first enzyme: Sll1336) and an L-arginine oxidase/dehydrogenase pathway (first enzyme: Slr0782) were high whether L-arginine or nitrate was the N-source, suggesting that these two pathways are the major L-arginine-degrading pathways and that they are expressed constitutively. The only exception is the carbamate kinase, whose transcript was found at elevated levels in L-arginine-grown cells. The lack of a substantial up-regulation of these transcripts, when cells were transferred from a nitrate-containing medium to an L-arginine-containing medium and an illumination of 50 μmol photons m^-2 ^s^-1 ^light, suggests that these pathways, besides having a function in the utilization of extracellular L-arginine, have a role in the complex dynamic metabolism of cyanophycin, which is not yet fully understood [8]. Such a functional L-arginine deiminase pathway would account for the products of L-arginine degradation identified in *Synechocystis *sp. PCC 6803 [51]. The bioinformatic evaluation in combination with the transcript analysis suggests that *Synechocystis *sp. PCC 6803 has an unusual L-arginine deiminase and an unusual L-arginine oxidase/dehydrogenase as the major L-arginine-degrading enzymes. An extended biochemical investigation of these two enzymes and the corresponding pathways is required before a statement can be made on how these two pathways are integrated in the overall C- and N-metabolism in *Synechocystis *sp. PCC 6803.

## Methods

### Bioinformatic analyses and tools for the interpretation of genomic DNA sequences

Bacterial genome sequences were obtained from the Kyoto Encyclopedia of Genes and Genomes database (KEGG). Database searches and similarity searches were done as described in Rueckert *et al.*[58] with nucleotide and amino acid sequences using the BlastN- and BlastP-algorithms [59]. Multiple sequence alignments were performed using the DIALIGN2 software [60]. The phylogenetic trees were calculated using the neighbor-joining method [61], which is integrated in the ClustalX software package [62]. The results were visualized as a radial tree with the interactive phylogenetic tree plotting program TreeTool [63].

### Cyanobacterial strains, growth conditions, and cell harvest

*Synechocystis *sp. strain PCC 6803 was obtained from the Pasteur Culture Collection of Cyanobacterial Strains, Paris, France. Cells were grown in gas wash bottles with a capacity of 250 ml in a stream of 2% carbon dioxide in air at 30°C. Growth either with nitrate or L-arginine as sole nitrogen source was performed basically according to Stephan *et al.*[10] except that the light intensity has been reduced from 200 to 50 μmol photons m^-2 ^s^-1^. Under these conditions the *Synechocystis *sp. PCC 6803 can grow with L-arginine without a stress phenotype. The standard inoculation corresponded to an absorbance of 0.3 at 750 nm (OD_750 nm_). Growth was determined as OD_750 nm _of *Synechocystis *sp. PCC 6803 cultures. After 24, 48, and 72 h cells were mixed 1:1 with crushed ice and harvested by centrifugation for 5 min at 4.000 × g in a table top centrifuge. Isolation of total RNA was performed as described previously [64] combined with an on-column DNase digestion step with the RNase-free DNase set from Qiagen (Qiagen, Hilden, Germany).

### Quantification of steady-state mRNA pools of selected transcripts with slot-blot RNA hybridization analysis

For slot-blot RNA hybridization experiments, 5 μg RNA were denatured for 10 min at 68°C in a formaldehyde/formamide-containing buffer and transferred to HybondN^+ ^membranes (Amersham Pharmacia Biotech, Freiburg, Germany) using the BioRad-Dot-blot SF Microfiltration Apparatus (BioRad) as described in the corresponding manual. RNA was UV cross-linked to the membrane and samples were probed with different PCR-derived digoxygenin-dUTP (Dig-dUTP) labeled gene-specific DNA probes (Table 14). Slot-blot RNA detection were performed using the CDP-Star ready-to-use system (Roche, Mannheim, Germany) according to the manufacturer's recommendation. The *rnpB *probe was used in all experiments to ensure equal loading.

**Table 14:** Primers used for amplification of gene-specific DNA probes for slot-blot RNA hybridization.

**Primer**	**Name**	**Amplified product**	**DNA sequence 5'→3' direction**
***sll1336***	***sll1336***	1686 bps	ATGTCGTACTGAGTCGCTTC TGGAGTGCAACATGCTGGAC
***sll0902***	***sll0902***	627 bps	TCCTTCACCGCGGCCATGTA CGGCAGACAGTGGAGCACAA
***slr1476***	***slr1476***	986 bps	GGTGGCCAGTTGGACTCGAA ATTCCTGAACAGTGCCTAGC
***sll0573***	***sll0573***	491 bps	AACGGAAGGCATGATCGGTT AACAGTGAGCGTAGTTGGTG
***slr0782***	***slr0782***	1325 bps	CCATCCTCGTCCTGTGATTG CCAGTACGAATTGCACCATC
***sll1077***	***speB2***	1054 bps	CAGCAGGAGGTTGACCAAGG CAGCATGGATATAGGCCGGT
***slr1022***	***argD***	1224 bps	GTTGTTGAATCCGTCGAAGC TTCTGCTTCCGTCACCACTA
***slr0370***	***gabD***	895 bps	GCCGAGGAATACTTAGCCGA GGTTAGTTGTCCATGCACTG
***sll1683***	***sll1683***	858 bps	ACCTCTTCCAAGCTGATCTG AGGCAGTGACATCGACGGTA
***slr0662***	***slr0662***	739 bps	GTTGGACCATTGACGACAGC CTGTCCAACATATCAGCTCG
***slr1312***	***slr1312***	853 bps	GCCTCCTGGAGCATTGAAGA CCAGCTTGACCAATTCCACA
***slr1469***	***rnpB***	599 bps	GCGGCCTATGGCTCTAATCA TTGACAGCATGCCACTGGAC

## Authors' contributions

SS performed the bioinformatic and the transcript analyses. CR aided the bioinformatic analyses and performed the phylogenetic analyses. EKP provided the knowledge and expertise on L-arginine catabolism and in part wrote the paper. KPM supervised the research and provided tables and figures. DS and all other authors have read and approved the final manuscript.
